# Mechanism of Salt-Induced Self-Compatibility Dissected by Comparative Proteomic Analysis in *Brassica napus* L.

**DOI:** 10.3390/ijms19061652

**Published:** 2018-06-03

**Authors:** Yong Yang, Zhiquan Liu, Tong Zhang, Guilong Zhou, Zhiqiang Duan, Bing Li, Shengwei Dou, Xiaomei Liang, Jinxing Tu, Jinxiong Shen, Bin Yi, Tingdong Fu, Cheng Dai, Chaozhi Ma

**Affiliations:** National Key Laboratory of Crop Genetic Improvement, National Center of Rapeseed Improvement in Wuhan, Huazhong Agricultural University, Wuhan 430070, China; yongyang218@163.com (Y.Y.); lzq0826@163.com (Z.L.); tongtong_1024@webmail.hzau.edu.cn (T.Z.); zhouguilong@webmail.hzau.edu.cn (G.Z.); looveduan@webmail.hzau.edu.cn (Z.D.); blihzau@163.com (B.L.); doushengwei@webmail.hzau.edu.cn (S.D.); xmliang1991@163.com (X.L.); tujx@mail.hzau.edu.cn (J.T.); jxshen@mail.hzau.edu.cn (J.S.); yibin@mail.hzau.edu.cn (B.Y.); futing@mail.hzau.edu.cn (T.F.)

**Keywords:** *Brassica napus*, iTRAQ, self-incompatibility, self-compatibility, stigma, salt

## Abstract

Self-incompatibility (SI) in plants genetically prevents self-fertilization to promote outcrossing and genetic diversity. Its hybrids in *Brassica* have been widely cultivated due to the propagation of SI lines by spraying a salt solution. We demonstrated that suppression of *Brassica napus* SI from edible salt solution treatment was ascribed to sodium chloride and independent of *S* haplotypes, but it did not obviously change the expression of SI**-**related genes. Using the isobaric tags for relative and absolute quantitation (iTRAQ) technique, we identified 885 differentially accumulated proteins (DAPs) in *Brassica napus* stigmas of un-pollinated (UP), pollinated with compatible pollen (PC), pollinated with incompatible pollen (PI), and pollinated with incompatible pollen after edible salt solution treatment (NA). Of the 307 DAPs in NA/UP, 134 were unique and 94 were shared only with PC/UP. In PC and NA, some salt stress protein species, such as *glyoxalase I*, were induced, and these protein species were likely to participate in the self-compatibility (SC) pathway. Most of the identified protein species were related to metabolic pathways, biosynthesis of secondary metabolites, ribosome, and so on. A systematic analysis implied that salt treatment-overcoming SI in *B.* *napus* was likely conferred by at least five different physiological mechanisms: (i) the use of Ca^2+^ as signal molecule; (ii) loosening of the cell wall to allow pollen tube penetration; (iii) synthesis of compatibility factor protein species for pollen tube growth; (iv) depolymerization of microtubule networks to facilitate pollen tube movement; and (v) inhibition of protein degradation pathways to restrain the SI response.

## 1. Introduction

Canola (*Brassica napus*), a plant from the *Brassicaceae* family, is one of the most important edible oil-producing crops, and its F1 hybrids have been widely cultivated worldwide for many years. However, self-incompatibility (SI) has been used in canola hybrid breeding for a long time. *B. napus* (AACC, 2*n* = 38), a member of *Brassica*, was formed by natural allopolyploidization between *Brassica oleracea* (CC, 2*n* = 18) and *Brassica rapa* (AA, 2*n* = 20) [1]. Although cultivated *B. napus* is a self-compatible species, SI lines can be generated by interspecific hybridization between *B. oleracea* and *B. napus* [2] or resynthesis from *B. oleracea* and *B. rapa* [3]. However, a troublesome question is how to propagate SI lines. Self-incompatibility of *Brassica* can be overcome by using physical and chemical methods, such as CO_2_ gas (3–5%) treatment [4], high temperature treatment [5,6], and stigmatic chemical treatments (e.g., ether and KOH) [7,8]. Hu et al. (1983) reported for the first time that spraying a salt solution in the anthesis of *B. napus* can efficiently overcome SI [9]. It is easy to operate and low-cost, and thus has been used to propagate SI lines for producing hybrid seeds in *B. oleracea* and *B. rapa* for a long time, and recently in *B. napus*, which has sped up hybrid breeding by self-incompatibility in *Brassica*. Considering its significant economic contribution, it is necessary to understand the molecular mechanism of salt solution-overcoming SI. However, little is known, especially in *B. napus.*

Self-incompatibility is one of the most important mechanisms for maintaining species diversity and offspring vigor [10]. Self-incompatibility in *Brassicaceae* is controlled sporophytically by a single multiallelic locus called the *S* locus or *S*-haplotype [11]. *S*-locus glycoprotein (*SLG*) was first identified as an SI-related gene in stigma, and subsequently it was demonstrated that it was not the female S determinant that was required for full manifestation of the SI response [12]. The female determinant *S*-locus receptor kinase gene (*SRK*), and the male determinant *S*-locus protein 11 gene (*SP11*)/*S*-locus cysteine-rich protein gene (*SCR*) (thereafter, refer to *SCR*) are the two vital genes of the SI response [13]. Before self-pollen grains contacts the stigma, *SRK* was inhibited by the thioredoxin h proteins *THL1* and *THL2* [14,15]. Once self-pollen grains lands on the stigma, *SRK* is activated by *SCR*, accompanied by another *S*-locus cytoplasmic receptor kinase, M-locus protein kinase (*MLPK*) [16]. Through protein phosphorylation, activated *SRK* phosphorylates AMR-repeat containing 1 (*ARC1*), resulting in ubiquitination and proteasomal degradation of SC-related proteins, such as *Exo70A1*, which is a subunit of the exocyst consisting of eight subunits [17,18]. Recently, the remaining seven subunits, *SECRETORY3*(*SEC3*), *SEC5*, *SEC6*, *SEC8*, *SEC10*, *SEC15*, and *EXO84*, were tested as compatible factors to promote compatible pollen grain acceptance [19]. Although many researchers have paid close attention to the *ARC1* linear ubiquitin–proteasome pathway, much evidence has suggested that the SI response is likely to be more complex, and to contain multiple parallel pathways rather than only the *ARC1* linear ubiquitin–proteasome pathway [20]. However, little is known about the others.

In recent years, omics technologies have played an increasingly important role in uncovering the self-incompatibility mechanism in *B. napus*. Microarray analysis of *B. napus* SC/SI pollination stigmas revealed a rapid stigma senescence response following compatible pollination [21]. Time-course transcriptome analysis at different time points showed that pollen–stigma interaction completed 30 min after pollination, and that the incompatible response had complicated signal transduction networks [22]. Nineteen unique SI-regulated proteins were identified by two-dimensional difference gel electrophoresis analysis coupled with matrix-assisted laser desorption ionization/time of flight/MS [23]. *Glyoxalase I* (*GLO1*), one of the 19 proteins, was further detected by reverse genetics to be a stigmatic compatibility factor necessary for reproduction [24]. So far, the proteomics approaches in *B. napus* self-incompatibility response have been limited to 2D gel electrophoresis analysis [25]. Isobaric tags for relative and absolute quantitation (iTRAQ) coupled to liquid chromatography-quadrupole mass spectrometry (LC-MS/MS), as the rapid rise of the proteomics approach, allow direct quantification and comparison of the protein expression levels of different samples with high efficiency and accuracy [26], and are widely used in plant extractome [27,28]. Therefore, it can be expected to reveal the mechanisms of salt-induced self-compatibility in *B. napus*.

In this study, phenotypic observations of pollen tube elongation and pod seed-set were first performed, SI-related gene expression levels were checked by qPCR, and iTRAQ was carried out for self-incompatible pollination, salt-induced self-compatible pollination, and self-compatible pollination. Our aims were (i) to elucidate some characteristics of salt-induced self-compatibility, (ii) to dissect any effect of salt solution on self-incompatibility-related gene expression, and (iii) to identify differentially accumulated proteins (DAPs) induced by salt solution and their functional annotations. All our results provide detailed information on the breakdown of SI by salt solution and abundant molecular data on compatible/incompatible pollen–stigma interactions, which provide new insights into networks of pollen–stigma interaction signal transduction.

## 2. Results

### 2.1. Self-Compatibility Induced by Salt Solution

**Figure 1:**
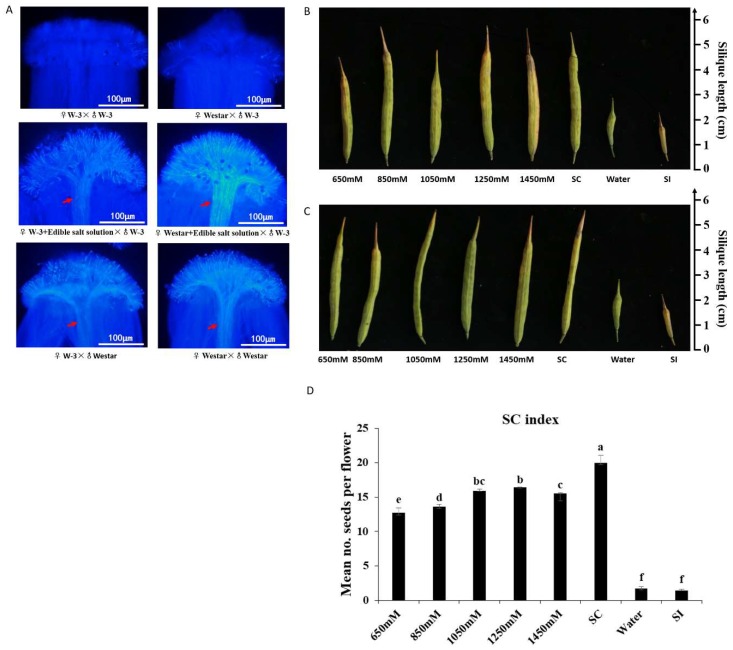
Breakdown of self-incompatibility by edible salt solution treatment in “W-3” and “Westar”. Pollination with “Westar” pollen grains was taken as a self-compatibility control (SC); self-incompatibility control was pollination with “W-3” pollen grains (SI), and pollination with “W-3” pollen grains after water treatment (Water); “Westar” flowers were emasculated one day before flower opening to avoid any contamination by self-pollen grains; concentrations of the salt solution were 650 mM, 850 mM, 1050 mM, 1250 mM, and 1450 mM. (**A**) Aniline blue assays performed 16 h after pollination with incompatible “W-3” pollen grains, compatible “Westar” pollen grains, and incompatible “W-3” pollen grains after 1050 mM edible salt solution treatment on the stigmas of both “W-3” and “Westar”. Every treatment included at least six individual pistils. Red arrows indicate abundant pollen tubes; (**B**) mature pods developed from “W-3” pistils that pollinated with self-pollen grains after treatment with edible salt solutions; (**C**) mature pods developed from “Westar” pistils were pollinated with “W-3” pollen grains after edible salt solution treatment; and (**D**) Mean self-compatibility indexes of “W-3” after edible salt solution treatment and their statistic test by one-way ANOVA with Scheffe post hoc tests. The different letters represent means that are significantly different at *p* < 0.05; Error bars indicate standard deviation.

On the stigmas of both “W-3” and “Westar”, few pollen grain adhesions and no pollen tube growth were observed when pollinated with “W-3” pollen grains, and pollen tubes could traverse stigma when pollinated with “Westar” pollen grains (Figure 1A). Seed-setting had consistent results that pods set few seeds with pollination of “W-3” pollen grains, but normal seed-setting was observed when pollinated with “Westar” pollen grains (Figure 1B,C), confirming that “W-3” and “Westar” had the same self-incompatible stigmas, “Westar” pollen grains were self-compatible, but “W-3” pollen grains were self-incompatible to “W-3” and “Westar” stigmas.

The effect of edible salt solution on breaking self-incompatibility was significant. In that case, “W-3” and “Westar” stigmas were treated with a salt solution and then pollinated with self-incompatible “W-3” pollen grains, abundant pollen tubes traversed through stigmas (Figure 1A), and many seeds set were observed (Figure 1B,C). The self-compatibility index (SCI) of each salt solution treatment and the self-compatible control were over 12, which was much higher than the SCI of the self-incompatible controls (<2) (Figure 1D). Salt solution treatment was also carried out in “S-1300“ line, whose self-incompatibility is controlled by the recessive class II S haplotype *BnS-1300*, while the dominant class I S haplotype *BnS-1* controls the self-incompatibility of “W-3”. Like “W-3”, “S-1300” was observed to have self-pollen elongation, and set many seeds when pollinated with self-pollen grains after treatment with the salt solution (Figure S1 and S2). Therefore, it was evident that breakdown of self-incompatibility by salt solution treatment was independent of *S* haplotypes.

To detect any possible effects of other substances in the edible salt, a pure NaCl solution was tested. As expected, the pure NaCl solution effectively destroyed the self-incompatibility of “W-3”, and “S-1300” as well (Figure S2). Therefore, it was reported that the edible salt solution-overcoming SI was due to sodium chloride (NaCl).

### 2.2. Effect of Salt Solution Treatment on Self-Incompatibility-Related Gene

**Figure 2:**
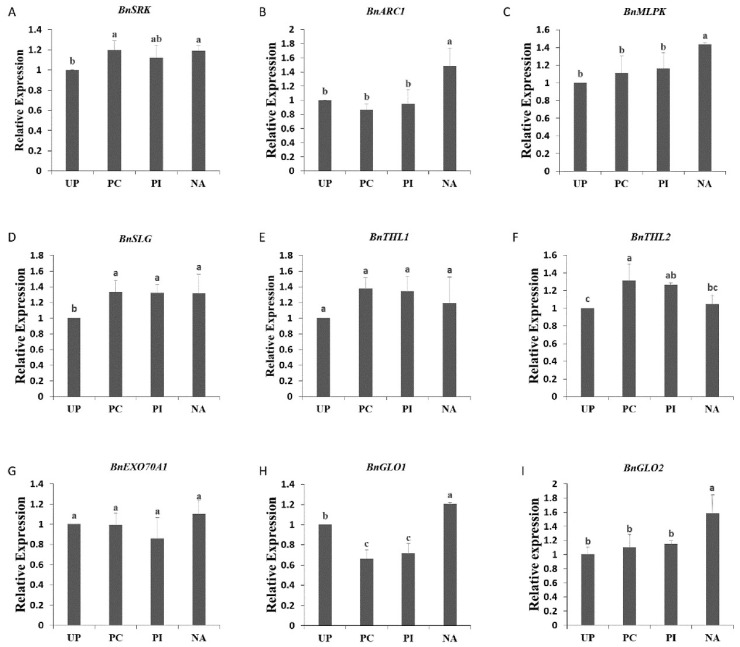
Expression analysis of SI-related genes, *BnSRK* (**A**), *BnARC1* (**B**), *BnMLPK* (**C**), *BnSLG* (**D**), *BnTHL1* (**E**), *BnTHL2* (**F**), *BnEXO70A1* (**G**), *BnGLO1* (**H**), and *BnGLO2* (**I**) in “Westar” stigmas. UP: unpollinated; PC: pollinated with “Westar” pollen grains; PI: pollinated with “W-3 pollen grains”; NA: pollinated with “W-3” pollen grains after edible salt solution treatment. The different letters are significantly different at *p* < 0.05. Error bars indicate standard deviation.

To detect whether salt-induced self-compatibility was due to SI/SC-related gene expression changes, qPCR was carried out to analyze the quantitative expression of *BnSRK*, *BnSLG BnEXO70A*1, *BnTHL*1, *BnARC*1, *BnMLPK, BnGLO1*, and *BnGLO2* (Figure 2). The four analyzed samples were “Westar” stigmas pollinated with no pollen grains (UP), pollinated with “Westar” pollen grains (PC), pollinated with “W-3” pollen grains (PI), and pollinated with “W-3” pollen grains after edible salt solution (1050 mM) treatment (NA). *BnSRK* and *BnSLG* were expressed at a higher level in NA than in UP, but there were no significant differences among PC, PI, and NA (Figure 2A,D). Both *BnEXO70A*1 and *BnTHL*1 showed similar gene expression among all four samples (Figure 2E,G). *BnTHL*2 gene expression revealed no change in NA compared with UP (Figure 2F). NA had the highest significant gene expression levels of *BnARC*1, *BnMLPK, BnGLO1*, and *BnGLO2* (Figure 2B,C,H,I). Both *BnGLO1* (*GSBRNA2T00047345001*) and *BnGLO2* (*GSBRNA2T00100627001*) were *glyoxalase I*, with higher homology to *AT1G11840* (*ATGLYI3*) and *AT1G08110* (*ATGLYI2*), respectively, but the homology was very low among them (data not shown), which suggested that there existed more than one *glyoxalase I* gene participating in the SC response. As *BnARC*1 is a positive self-incompatibility factor and *BnEXO70A*1 and *BnGLO*1 are negative self-incompatibility factors, expression changes of SI-related genes could not explain the mechanism of edible salt solution-overcoming SI.

In the following proteomics results, we identified *SRK* (*GSBRNA2T00102188001*) protein, but no quantitative data were acquired (Table S1). Although *SLG* (*GSBRNA2T00102191001*), *THL2* (*GSBRNA2T00043000001*) and *GLO1* proteins were identified, their expression was not significantly changed based on screening criteria. Another *glyoxalase I* gene, *GLO2*, comparing with UP, was increased by ~1.55 and ~1.82 fold in PC and NA, respectively. However, other SI-related genes, *ARC1* (*GSBRNA2T00069618001*), *MLPK* (*GSBRNA2T00101663001*), *EXO70A1* (*GSBRNA2T00136453001*), and *THL1* (*GSBRNA2T00076902001*) were not identified in our study. These results indicated that suppression of SI by salt solution was because of increasing SC-related protein species accumulation.

### 2.3. Quantitative Identification of Proteins Using iTRAQ

iTRAQ analysis was conducted to detect any mechanism of salt-induced self-compatibility. The four samples for iTRAQ analysis were the same with those for qPCR analysis described above (Figure S3). After merging data from the two replicates, a total of 401,216 spectra were generated from the four samples. Using the search engine of Mascot (version 2.3.02, Boston, MA USA), we obtained 30,805 unique spectra, 17,734 identified peptides including 11,949 unique peptides and 5458 identified protein species (Figure S4A, Table S1).

For the 5458 identified protein species, the protein mass from 10 to 100 kDa were approximately normally distributed, and the percentages of 10–50, 50–100, and above 100 kDa protein were 47%, 40%, and 13%, respectively (Figure S4B). The peptide lengths of the identified protein species were mainly distributed in the 7–17 amino acid range (Figure S4C). The peptide number distribution (Figure S4D) of 1 single peptide, 2−5 peptides, 6−10 peptides, and >11 peptide proteins comprise 2811, 2316, 286, and 45, respectively. For the distribution of protein sequence coverage, 40−100%, 30−40%, 20−30%, 10−20%, and under 10% variation in protein sequence coverage accounted for 5.55%, 6.97%, 13.59%, 24.99%, and 48.99% coverage, respectively (Figure S5).

To categorize the identified 5458 protein species, gene ontology (GO) categories were conducted using the Blast2GO program (Available online: http://www.gene-ontology.org) against the non-redundant protein database (NR; NCBI). They possessed a wide range of biological processes, cellular components, and molecular functions, which could be grouped into 22, 15, and 16 categories, respectively (Figure S6). In terms of biological processes, the top three groups were cellular process (13.86%), metabolic process (13.57%), and single-organism process (10.53%). The primary cellular components were cell (22.12%), cell part (22.12%), organelle (18.13%), and membrane (9.49%). For the molecular function of these proteins, binding (43.94%), catalytic activity (41.10%), structural molecule activity (3.64%), and transporter activity (3.33%) were mainly included. To complement the GO category, COG enrichment analysis was performed according to the sequence of proteins. The identified 5458 protein species showed differential enrichment for post-translational modification, protein turnover, and chaperones, carbohydrate transport and metabolism, translation, ribosomal structure and biogenesis, and energy production and conversion (Figure S7). The above results showed that all 5458 identified protein species involved a wide range of cellular components, molecular functions, biological processes, and functional categories.

### 2.4. Differentially Accumulated Proteins

**Figure 3:**
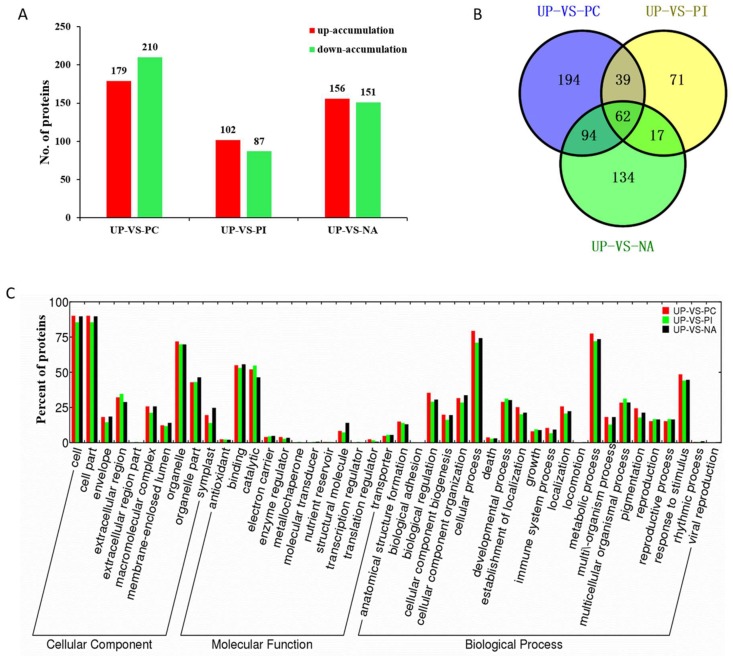
Functional classification of differentially accumulated proteins (DAPs) in UP-VS-PC, UP-VS-PI, and UP-VS-NA. (**A**) The numbers of DAPs; (**B**) A Venn diagram of DAPs showing unique and shared protein species; and (**C**) The cellular component, molecular function and biological process of DAPs.

Differentially accumulated proteins (DAPs) were considered as those displayed a fold change >1.3 or <0.77 in relative abundance and a *p* value < 0.05. Compared with UP, 389, 189, and 307 protein species exhibited a differentially accumulated pattern, with 179, 102, and 156 protein species up-accumulated and 210, 87, and 151 protein species down-accumulated in PC, PI, and NA, respectively (Figure 3A, Table S2).

A Venn diagram was generated to show the relationship of DAPs among PC/UP, PI/UP, and NA/UP (Figure 3B, Table S3). In total, 194, 71, and 134 protein species showed unique differential accumulation in PC/UP, PI/UP, and NA/UP, respectively. Of the DAPs, 101, 79, and 156 protein species were shared by PC/UP and PI/UP, PI/UP, and NA/UP, and PC/UP and NA/UP, respectively. PC/UP, PI/UP, and NA/UP had 62 common DAPs, showing that both self-incompatible and self-compatible responses require some proteins as basic biological actions. Cluster analysis of the 62 DAPs indicated that PC and NA had a similar protein expression pattern (Figure S8). Fewer unique DAPs in PI/UP and more shared DAPs by PC/UP and NA/UP indicated that self-compatibility response induced more protein changes, and was thus more complicated than the self-incompatibility response.

Out of the 307 differentially accumulated proteins in NA/UP, 17 were shared only with PI/UP and 94 shared only with PC/UP, in addition to the 62 common and 134 unique DAPs. Eighty-nine of the 94 protein species displayed the same expression trend in PC/UP and NA/UP (45 up-accumulated and 44 down-accumulated) (Table S3). We observed that 94 were shared with PC/UP and 134 of all 307 DAPs were unique in NA/UP, indicating that salt-induced self-compatibility might be attributed partly to the commonly changed protein species between PC/UP and NA/UP, and partly to unique protein species.

### 2.5. Functional Classification of the Differentially Accumulated Proteins

Blast2go software was used to classify and group protein species based on their GO annotations. A total of 373 DAPs (95.9%) in PC/UP were classified into 41 functional groups (Figure 3C); the biological processes, cellular components, and molecular functions accounted for 22, 11, and 8 GO terms, respectively. GO enrichment analysis showed that these proteins were significantly enriched in the GO categories “cellular macromolecular complex assembly” (*p* = 2.39 × 10^−6^), “macromolecular complex assembly” (*p* = 1.41 × 10^−5^), “generation of precursor metabolites and energy” (*p* = 2.55 × 10^−5^), “photosynthesis, light reaction” (*p* = 6.31 × 10^−5^), “ribosome biogenesis” (*p* = 6.48 × 10^−5^), “ribonucleoprotein complex biogenesis” (*p* = 9.28 × 10^−5^), “photosystem II assembly” (*p* = 1.08 × 10^−4^), and “monosaccharide metabolic process” (*p* = 1.72 × 10^−4^) in biological processes (Table S4).

A total of 291 DAPs (94.8%) in NA/UP were classified into 42 functional groups (Figure 3C), among which biological processes, cellular components, and molecular functions accounted for 21, 11, and 10 GO terms, respectively. All annotated proteins were significantly enriched in the GO categories “RNA methylation” (*p* = 1.14 × 10^−7^), “translation” (*p* = 1.47 × 10^−6^), “RNA modification” (*p* = 2.61 × 10^−5^), “response to cold” (*p* = 1.01 × 10^−4^), “response to temperature stimulus” (*p* = 1.35 × 10^−4^), “plant-type cell wall organization” (*p* = 3.49 × 10^−4^), “defense response, incompatible interaction” (*p* = 3.52 × 10^−4^), “plant-type cell wall modification” (*p* = 3.70 × 10^−4^) in biological processes (Table S4).

For PI/UP, 179 identified DAPs (94.7%) were classified into 44 functional groups (Figure 3C), of which biological processes, cellular components and molecular functions accounted for 20, 12, and 12 GO terms, respectively. These proteins were significantly enriched in the GO categories “plant-type cell wall modification” (*p* = 4.59–5), “plant-type cell wall organization” (*p* = 8.51 × 10^−4^), “cell wall modification” (*p* = 9.30 × 10^−4^), “external encapsulating structure organization” (*p* = 0.0017), “oxidation–reduction process” (*p* = 0.0019), “fertilization” (*p* = 0.0032), “double fertilization forming a zygote and endosperm” (*p* = 0.0032), “pollen exine formation” (*p* = 0.0044) in biological processes (Table S4).

**Figure 4:**
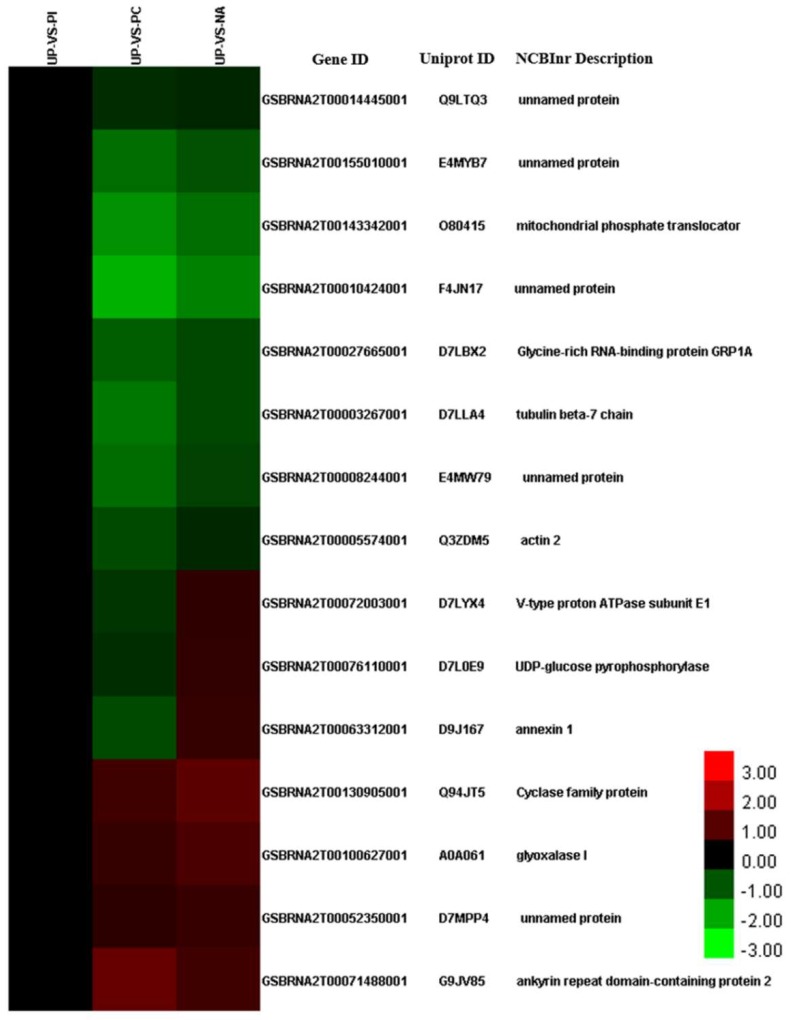
Cluster analysis of DAPs enriched in response to salt stress between UP-VS-PC, UP-VS-PI, and UP-VS-NA. The detailed information of these DAPs is listed in Table S6.

The 94 DAPs shared by NA/UP with only PC/UP contained 38 GO categories, of which 9, 9, and 18 categories were included in biological processes, cellular components, and molecular functions, respectively (Figure S9). GO enrichment analysis revealed that the 94 proteins were significantly enriched in “translation” (*p* = 1.05 × 10^−7^), “RNA methylation” (*p* = 3.38 × 10^−6^), “RNA modification” (*p* = 5.97 × 10^−5^), “outer mitochondrial membrane organization” (*p* = 3.27 × 10^−4^), “protein import into mitochondrial outer membrane” (*p* = 3.27 × 10^−4^), “macromolecule methylation” (*p* = 5.20 × 10^−4^), “ribosome biogenesis” (*p* = 8.89 × 10^−4^), and “ribonucleoprotein complex biogenesis” (*p* = 0.0010) in the biological processes (Table S5). Although “response to salt stress” (*p* = 0.16028) was not a significantly enriched term based on a cutoff value of a *p* < 0.05 threshold, 15 proteins were involved in “response to salt stress”, and all of these proteins were DAPs in PC/UP and NA/UP, rather than in PI/UP (Figure 4), indicating that these protein species were likely to be targets of edible salt solution-overcoming SI.

### 2.6. Primary Metabolic Pathways of Differentially Accumulated Proteins

**Figure 5:**
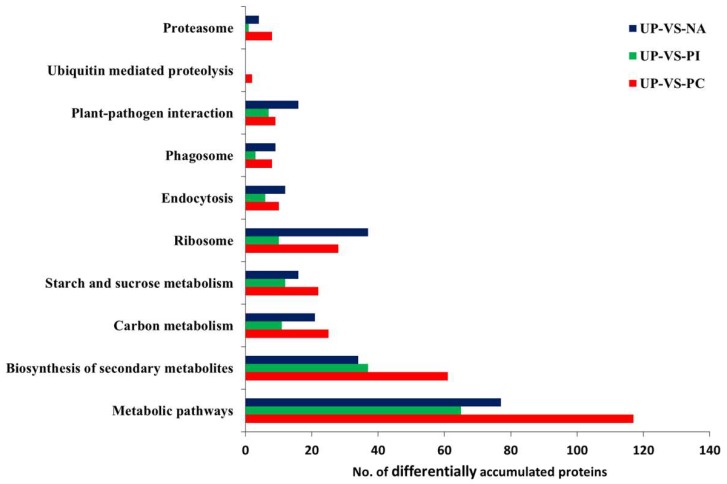
The number of DAPs involved in representative pathways in UP-VS-PC, UP-VS-PI, and UP-VS-NA. Detailed information for these DAPs is listed in Table S9.

All DAPs were classified based on the KEGG database. They were involved in comprehensive metabolic pathways (Figure 5, Table S7). As ribosome, endocytosis, proteasome, plant–pathogen interaction, phagosome and ubiquitin-mediated proteolysis pathways were suggested to be involved in self-incompatible/compatible responses [20,29,30,31,32], DAPs clustered in these pathways were shown in Figure 5. Compared with PI/UP, both PC/UP and NA/UP had more DAPs in these pathways, except ubiquitin-mediated proteolysis, showing that ribosome, endocytosis, proteasome, plant–pathogen interaction, and phagosome pathways might be involved in salt-induced self-compatible responses.

There were seven significant pathways for 194 specifically expressed protein species in PC/UP, including photosynthesis, amino sugar and nucleotide sugar metabolism, proteasome, metabolic pathways, cyanoamino acid metabolism, oxidative phosphorylation, and carbon fixation in photosynthetic organisms (Table 1, Table S8). However, PI/UP and NA/UP had only two and four significant pathways for specifically expressed protein species, respectively. In the specific DAPs of PI/UP, inositol phosphate metabolism, and biosynthesis of secondary metabolites, were the two significant pathways. The ascorbate and aldarate metabolism, ribosome, plant–pathogen interaction, and phagosome were present for 134 specifically expressed protein species in NA/UP. KEGG enrichment analysis indicated that PC, PI, and NA induced uniquely expressed protein species and diverse pathways.

The 62 common DAPs among PC/UP, PI/UP, and NA/UP were mainly enriched in oxidative phosphorylation, photosynthesis, valine, leucine, and isoleucine degradation, and plant–pathogen interaction; out of these, three pathways were related to energy metabolism. Thus, we considered that pollen–stigma interaction was an energy-consuming process.

**Figure 6:**
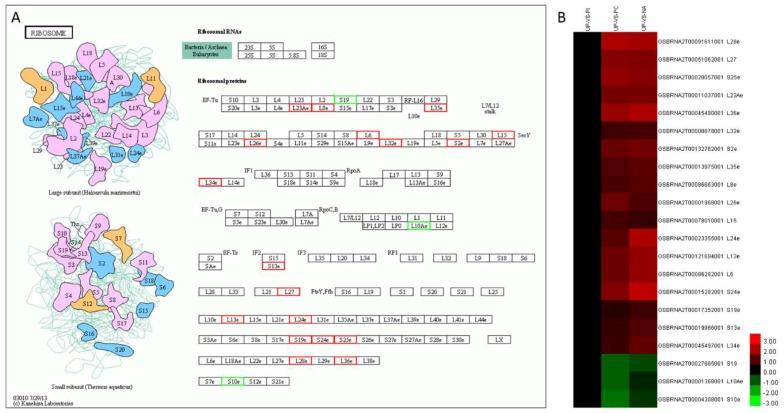
The overlapping differentially accumulated proteins between UP-VS-PC and UP-VS-NA enriched in the ribosome. (**A**) Twenty-one proteins mapped to ribosome, red box indicates up-accumulated protein, green box indicates down-accumulated proteins; and (**B**) heat map of the 21 differentially accumulated proteins; red means up-accumulated and green means down-accumulated.

Ribosome and phagosome were two significant enrichment pathways of the common 94 DAPs only between PC/UP and NA/UP. The ribosome pathway included 21 protein species, of which 18 protein species were commonly up-accumulated, and three were down-accumulated (Figure 6A,B). There were four protein species of the 94 DAPs enriched in phagosome, three protein species showed the same changed trend (one upregulation and two downregulations), while the remaining one was up-accumulated in NA and down-accumulated in PC (Figure S10A,B).

### 2.7. Validation of Proteomics Data by Quantitative Real-Time RT-PCR

**Figure 7:**
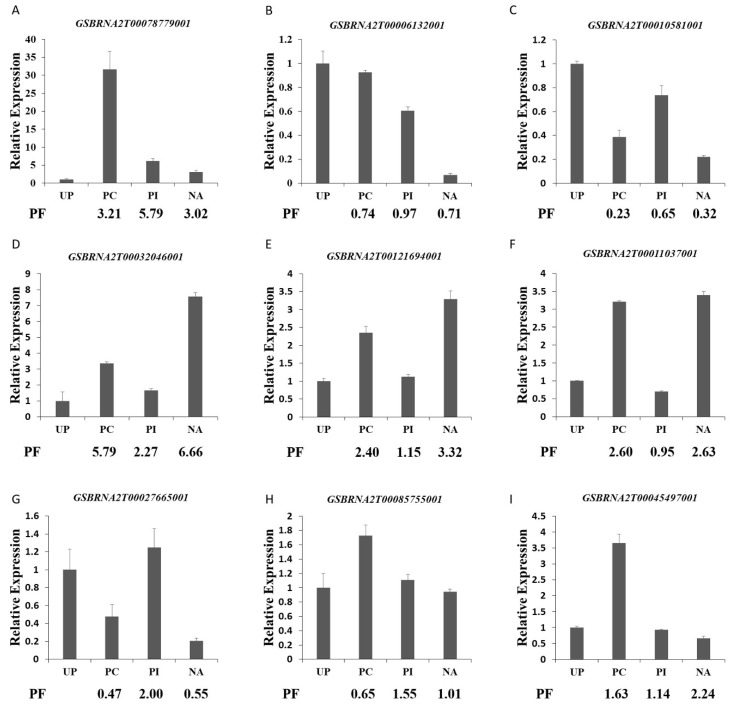
qPCR analysis of the genes selected randomly from differentially accumulated proteins. (**A**) *GSBRNA2T00078779001*; (**B**) *GSBRNA2T00006132001*; (**C**) *GSBRNA2T00010581001*; (**D**) *GSBRNA2T00032046001*; (**E**) *GSBRNA2T00121694001*; (**F**) *GSBRNA2T00011037001*; (**G**) *GSBRNA2T00027665001*; (**H**) *GSBRNA2T00085755001*; (**I**) *GSBRNA2T00045497001*; PF, proteomic folds of UP-VS-PC, UP-VS-PI, and UP-VS-NA. Error bars indicate standard deviation.

To verify the conformance between the mRNA expression level and the abundance of protein species, expression analysis of nine protein species selected randomly from the DAPs were performed by qPCR (Figure 7). The expression levels of seven genes exhibited the same trend as the expression abundance of the corresponding protein species, such as *GSBRNA2T00078779001*, *GSBRNA2T00006132001*, and *GSBRNA2T00010581001*. In addition, the expression level of the remaining two genes displayed an inconsistent trend with the abundance of their corresponding protein species. The nonconformity between the expression level of the two genes and the abundance of corresponding protein species was likely due to diverse posttranslational modifications, such as protein phosphorylation, ubiquitination, and glycosylation, after different pollination treatments. Most of the previous studies reported that the consistency between mRNA and protein expression is very low, and mRNA levels can only explain 27~40% of protein level variation [33]. So, the mechanism of pollen–stigma interactions could be well understood by proteomics technology.

## 3. Discussion

### 3.1. Characteristics of Edible Salt Solution Destroyed SI in B. napus

Propagating SI lines is basic and necessary for the utilization of self-incompatibility in hybrid seed production. To obtain many self-incompatible seed lines, a double-cross model previously in *Brassica* [34] and a triple-cross model in *B. napus* [35] were proposed. In the triple-cross model, a self-incompatible line was generated by crossing a maintainer line with an SI line; a three-component hybrid was developed through pollinating SI line with a restorer line. However, not enough hybrids were produced because of the lack of an effective, low-cost, and simple method for amplifying SI lines. Spraying an edible salt solution to overcome SI in *Brassica* species had been reported by Hu et al. in 1980s, since then it had been used to propagate SI lines. These authors proposed that the breakdown of self-incompatibility resulted from disintegrating of callose on stigmas, which was considered as substance to prevent self-pollen tubes elongation. In this study, phenotypic observations of pollen tube elongation and pod seed-set showed that 650~1450 mM edible salt solution could significantly break the self-incompatibility of *B. napus*. The overcoming of SI was due to NaCl, and it was independent of *S* haplotypes. Gene expression analysis by qPCR showed no relationship between SI-related gene expression changes and overcoming SI by edible salt solution treatment. The iTRAQ technique identified 307 differentially accumulated proteins (DAPs) in NA/UP, with 134 unique and 94 shared only with PC/UP. Functional classification showed that ribosome and phagosome were two significant enrichment pathways at the 94 DAPs. All the results indicated that SI destroyed by edible salt solution treatment was attributed to the protein species of self-compatible pollination response and to unique protein species, and the DAPs in the ribosome and phagosome pathways might be candidates induced by salt solution.

We identified 307 differentially accumulated proteins (DAPs) in NA/UP by iTRAQ technology, much more than the 19 DAPs by 2-D difference gel electrophoresis analysis [23]. Furthermore, we could compare DAPs in NA/UP with DAPs in PC/UP because of our specific materials, “W-3” and “Westar”. “Westar” and “W-3”, used in this research, had the same self-incompatible stigmas because “W-3” was only developed via genetic transformation, complementing the normal function of male determinant gene *BnSP11-1* in self-compatible “Westar” pollen grains. We sampled stigmas of “Westar” rather than “W-3” because “Westar” produced many self-pollinated seeds, and did not have any gene transformation, most importantly, DAPs in NA/UP could accurately be compared with those in PC/UP. Out of the 19 proteins, glyoxalase I (GLO1) was confirmed to be a stigmatic compatibility factor [24]. Therefore, it could be expected that knockout by CRISPR (clustered regularly interspaced short palindromic repeats) or overexpression by transformation of the DAPs in NA/UP could be used to identify genes responsible for edible salt solution-induced self-compatibility.

### 3.2. Salt Stress and Salt-Induced Self-Compatibility

In the present study, we used concentrated salt which led us to propose that salt stress was involved in salt-induced self-compatibility. Among the 94 common DAPs between PC/UP and NA/UP, 15 proteins, including actin 2, tubulin beta-7, annexin 1, glyoxalase I, UDP-glucose pyrophosphorylase, and H^+^-ATPase, were enriched in response to salt stress (Figure 4, Table S6). As an important component of the plant cytoskeleton, actin filaments (AFs) and microtubules (MTs) take part in various biological processes, such as cell division, intracellular trafficking, cell shape maintenance, and respond to different stresses, including salt stress. A long-term observational study showed that salt stress induced AF assembly and bundle formation [36,37], and polymerization of actin in the stigmatic papilla following SC pollination [23,38]. However, Dearnaley et al. (1999) suggested that neither compatible nor self-incompatible pollinations were involved in reorganization of the papillar cytoskeleton at the early stage in *B. napus* [39]. We observed reduced expression of actin 2 in both PC/UP and NA/UP. Therefore, the function of AFs on edible salt solution overcoming SI needs to be further studied.

A high salt concentration could depolymerize the polymerized MTs [40,41], and the localized depolymerization of MTs was observed after self-compatible pollination [23]. Tubulin beta-7, a subunit of the MT network, was down-accumulated in PC/UP and NA/UP, which was consistent with these studies. Darya et al. (2013) demonstrated that vesicle-mediated activity was rapidly induced in stigmatic papillae by compatible pollen grains in the *Brassicaceae* [40], and annexin 1, which is involved in Golgi-mediated secretion, was up-accumulated in NA/UP, suggesting that exocytosis likely participates in salt-induced self-compatibility. Glyoxalase I (*GLOI*) was identified as a compatibility factor in *B. napus* [24]; overexpression of *GLOI* from *Brassica juncea* was considered to confer salinity tolerance in transgenic tobacco [41]. *BnGLO2* (*GSBRNA2T00100627001*) as glyoxalase I was identified and up-accumulated in PC/UP and NA/UP (Table S6), and qPCR also showed it was upregulated in NA/UP (Figure 3E), which were consistent with it being a compatibility factor. It was proposed that salt stress increased the expression of *GLOI* and then led the SC response to occur. UDP-glucose pyrophosphorylase (*UGPase*) is a ubiquitous enzyme in plants, and produces UDP-glucose, which is essential for sucrose and polysaccharide synthesis. Studies by *UGPase* mutants revealed loss or drastically decreased activity of *UGPase*, resulting in a decreased number of seeds produced [42]. The increased expression of *UGPase* in NA/UP indicated that salt solution treatment could promote seed production in our study. Salt stress can induce osmotic stress and ion stress [43]; ion transport is necessary for maintaining ionic balance. *H^+^-ATPase* is a proton pump that uses the energy from ATP hydrolysis to produce a proton gradient, which can maintain ion balance. It has already been proven to be an essential component of key signal transduction [44]. After SC pollination, the *H^+^-ATPase* protein was significantly down-accumulated. By contrast, salt solution treatment induced *H^+^-ATPase* up-accumulation, which was to maintain ion balance or promote SC response in stigmas. In conclusion, salt stress-related proteins might be helpful for salt-induced self-compatibility.

### 3.3. Protein Biosynthesis and Salt-Induced Self-Compatibility

We found that PC and the exogenous application of edible salt solution induced abundant DAPs in stigmas of *B. napus*. There were 389, 189, and 307 DAPs in PC/UP, PI/UP, and NA/UP, respectively (Figure 6A). The number of DAPs of PC/UP and NA/UP were obviously greater than PI/UP. Salt solution treatment changed the expression pattern of DAPs, as implied by the clustering analysis of 62 commonly DAPs (Figure S8). PC/UP and NA/UP possessed more increased DAPs mapping on the ribosome than PI (Figure 6), which suggested that salt solution treatment regulated abundant protein expression, and might play a role in salt solution overcoming SI.

The site of protein biosynthesis is the ribosome, which supports organism development, certainly including pollen–stigma interaction. Both rRNAs and ribosomal proteins execute ribosome biosynthesis [45]. The 94 common DAPs between PC/UP and NA/UP were significantly enriched in the ribosome pathway (Figure 6). A total of 21 differentially accumulated ribosomal protein species were identified, 18 of which were commonly up-accumulated, and only 3 down-accumulated in PC/UP and NA/UP. There were thirteen up-accumulated large subunit ribosomal proteins, including *L6*, *L15*, *L27*, *L36*, *L8e*, *L13e*, *L23Ae*, *L24e*, *L26e*, *L28e*, *L32e*, *L34e*, and *L35e*, and 4 small subunit proteins, *S2e*, *S13e*, *S19e*, *S24e*, and *S25e*. The down-accumulated ribosomal proteins included two small subunit proteins, *S10e* and *S19*, and one large subunit protein, *L10Ae*. These results indicated that ribosomal proteins, including large and small subunits, were important factor during the pollen–stigma interaction of PC and NA.

When the pollen grain lands on the stigma, hydration rapidly happens, which causes the pollen grains to germinate and pollen tubes to emerge. To penetrate the stigmatic papilla, the pollen tubes grow through the stigmatic cuticle, and then enter the outer layer of the stigmatic cell wall [46,47]. At this moment, stigmatic cell wall modification is required, which is dependent of proteins secreted by the stigmatic papilla. *Exo70A1*, as a part of the exocyst complex, might be required for the delivery of proteins required for pollen tube growth through the stigmatic cuticle and cell wall. In our study, abundant ribosome proteins were identified in PC and NA, suggesting that pollen tube growth-related proteins were synthesized through ribosomes in stigma. The reason why salt solution treatment overcame SI was probably due to the dynamic of protein synthesis in stigmas, which was necessary for pollen tubes growth.

### 3.4. Ubiquitin-Mediated Processes and Salt-Induced Self-Compatibility

Ubiquitination involves an intricate cascade process that is controlled by three enzyme families, including E1 (Ub-activating enzyme), E2 (Ub-conjugating enzyme), and E3 (Ub ligase), which are large extended gene families. There are 16 genes encoding E1s, 45 genes encoding E2s, and more than 1400 genes encoding E3s in *Arabidopsis thaliana*. E3 is an important factor that defines substrate specificity. Ubiquitin (Ub) protein, consisting of 76 amino acids, is highly conserved in all eukaryotic species. It covalently marks target proteins for degradation by the 26S proteasome in different organisms, which has been well discussed in mediating different cellular processes [48,49,50]. *ARC1*, as an E3 ligase, plays an important role in the SI of *Brassica* by targeting and transferring compatibility factors to the proteasome. In *B. napus*, 30 min after incompatible pollination, increased ubiquitination occurred, and the proteasome’s proteolytic pathway was essential for the SI response [17,23]. However, only two DAPs of PC/UP were involved in ubiquitin-mediated proteolysis in our study, and both were ubiquitin-conjugating enzyme E2, not E3, suggesting that ubiquitination had a steady state in PC, PI, and NA (Table S9). Additionally, a total of 12 DAPs were mapped on the proteasome pathway, of which 8, 1, and 4 DAPs belong to PC/UP, PI/UP, and NA/UP, respectively. The cytosolic 26S proteasome is used exclusively, which contains one 20S protein subunit and two 19S regulatory cap subunits [51]. Six DAPs were identified as proteasome subunits, two proteasome regulatory subunits were significantly up-accumulated (~2-fold), and one down-accumulated in NA/UP (Table S9). We considered that salt-induced self-compatibility was not a result of the inhibited ubiquitin-proteasome pathway.

### 3.5. Roles of Endocytosis and Phagosome in Salt-Induced Self-Compatibility

Self-incompatibility was more complex, involving pathways in addition to the *ARC1* linear ubiquitin–proteasome pathway, and edible salt solution-overcoming SI might affect these pathways. The two pathways of endocytosis and phagosomes, which are essential processes in cells to control the dynamics and turnover of plasma membrane proteins, were detected in the current study (Figure 5, Table S9). Endocytosis is a process by which cells internalize portions of their plasma membrane and extracellular substances via the formation of endosomes. Many receptors are internalized by endocytosis following the binding of corresponding ligands in eukaryotes. The two leucine-rich repeat receptor kinases flagellin-sensitive 2 (*FLS2*) and brassinosteroid-insensitive 1 (*BRI1*) were well studied and associated with endocytosis in *Arabidopsis thaliana*. Inactive *FLS2* localizes to the PM, whereas it is rapidly internalized and degraded upon binding to the *flg22* protein, which is a 22-amino acid peptide containing a conserved epitope from bacterial flagellin [52]. *BRI1* localizes both to the PM and cytoplasmic compartments, and its localization and trafficking are independent of its ligand [53]. Ivanov et al. (2009) demonstrated that *SCR*–*SRK* interaction took place at the plasma membrane, and that SRK moved into endosomes where the *SRK* negative regulator *THL1* was enriched, which was different from the *BRI1* signaling pathway, and was more similar to *FLS2* [32]. Clathrin-mediated endocytosis was the most prominent endocytic pathway in plants and animals. In animals, auxilin recruits the *ATPase HSC70* (heat shock cognate 70) to initiate the disassembly of the clathrin coat fusing with endosomes [54,55], and the clathrin-uncoating process is conserved in plants [56]. In the present study, *Hsc70-1* was identified as significantly down-accumulated in PC/UP and PI/UP, and conversely, *Hsc70-G8* was upregulated in NA/UP; these results suggested that salt-induced self-compatibility was likely to inhibit or degrade *SRK* by promoting fusion with endosomes (Table S9). *SRK* protein was not identified in NA/UP, probably because it was degraded. Additionally, six subtilases (*SBTs*), which are a family of subtilisin-like serine proteases [57,58], were identified in the endocytosis pathway. Although only one *SBT* was detected and up-accumulated in PI/UP (Table S9), we also thought that SI was likely to degrade compatibility factors by the *SBT* system because few proteins were also identified in the ubiquitin-26S proteasome system. Interestingly, four of six *SBTs* were significantly down-accumulated in NA/UP, and only one *SBT* was identified and down-accumulated in PC/UP (Table S9). These *SBTs* might be involved in the SI response to degrade compatibility factors. Therefore, we considered that salt-induced self-compatibility could maintain abundant compatibility-related protein expression by inhibiting subtilase expression.

Endocytosis includes pinocytosis and phagocytosis by phagocytosis to form an internal compartment known as a phagosome. Phagosomes are essential for tissue homeostasis and the innate immune response, and can fuse with lysosomes to degrade bacteria or apoptotic and senescent cells [59]. In phagosomes, four beta tubulins were detected, and three were down-accumulated in NA/UP (Table S9). As mentioned above, localized destabilization of the MT network occurred 30 min after compatible pollinations in *Brassica napus* [23], further suggesting that salt solution treatment decreased tubulin expression to promote the SC response. Calnexin and calreticulin were detected in the phagosome. There were 3 calnexins and 1 calreticulin, of which, 2 calnexins and 1 calreticulin were up-accumulated in NA/UP. Calnexin is a membrane-tethered homologue of the wholly lumenal calreticulin [60], and it is highly conserved in eukaryotes [61] and binds calcium ions that participate in protein folding. Salt stress could induce Ca^2+^ fluctuation [43], and Ca^2+^ is also involved in SI/SC in *Brassica* [62,63]. It is likely that compatibility factors assembled and transported to the PM by calnexin and calreticulin following edible salt solution treatment in stigmas. Additionally, abundant ribosomal proteins were synthesized in stigmas, by which compatibility factor proteins were possibly synthesized in a large quantity, then protein folding was performed by calnexin and calreticulin to promote the SC response in PC/UP and NA/UP. Therefore, we proposed that edible salt solution treatment induced the expression of proteins related to endocytosis and phagosomes to promote the SC response in stigmas of *B. napus*.

### 3.6. Plant–Pathogen Interaction and Pollen–Stigma Interaction

Many studies have suggested that the recognition response between pollen and stigma is similar to that of plant–pathogen interactions [29,30,31], but very little evidence has supported this conclusion. Recently, transcriptomic analysis of *B. napus* SC/SI provided more detailed molecular information about pollen–stigma interactions and plant–pathogen interactions [22]. In our study, 18 DAPs were involved in the plant–pathogen interaction pathway, of which the number of 16 DAPs in NA/UP was far greater than that in PC/UP and PI/UP (Figure 5, Table S9). Therefore, we speculated that there were some correlations between plant–pathogen interactions and pollen–stigma responses or salt-induced self-compatibility. When compatible pollen lands on stigma, it penetrates the cell wall of papillae cells, similarly to pathogens infecting a plant, which also need to pass through the cell wall. Obviously, both pathways must modify plant cells to promote pollen tubes or pathogens from entering the cell or tissue. Pectin is abundant in plant cell walls, and can be degraded or modified by pectin methylesterase, polygalacturonase (PG), and pectin lyase [64]. Extensins (*EXTs*), another important plant cell wall component, are a diverse family of hydroxyproline-rich glycoproteins (HRGPs), and are characterized by the repeated occurrence of serine (Ser) followed by several consecutive prolines (Pro) [65,66]. The mutual crosslinking properties of EXTs contribute to the extracellular matrix and play roles in plant development and defense responses [67,68]. In our study, three PG inhibitor proteins were detected in NA/UP and significantly down-accumulated (Table S9), and one leucine-rich repeat extensin-like protein 4 was identified and significantly down-accumulated in PC/UP and NA/UP, suggesting that edible salt treatment promoted degradation of the cell wall of stigma, consequently permitting pollen tubes enter into pistils.

It is assumed that plant SI and plant immunity processes may share the same basal genetic defense network, and genes involved in SI and defense might have common mechanisms [30]. In our study, and we identified some plant defense proteins, such as calmodulin, allergen, leucine-rich repeat (*LRR*) family protein, and peptidoglycan-binding LysM domain-containing protein. Ca^2+^ plays important signaling roles in the response to biotic and abiotic stress, including plant–pathogen interaction and salt stress. The Ca^2+^ signal is transduced through Ca^2+^ sensors, which include calmodulins (*CaMs*), CaM-like proteins (*CMLs*), Ca^2+^-dependent protein kinase (*CDPKs*), and calcineurin B-like proteins (*CBLs*). Ca^2+^ as a signaling molecule also participates in pollen–stigma interactions [62,63]. CaMs, as a target of Ca^2+^, were significantly up-accumulated in PC/UP, PI/UP and NA/UP, indicating that Ca^2+^ plays an important role in pollen–stigma interactions and salt-induced self-compatibility. Many resistance (R) proteins have a conserved *LRR* domain, which functions in some developmental and immune signaling pathways, and participates in receptor/co-receptor complex formation [69,70]. Of the three identified *LRR* proteins, two were down-accumulated in NA/UP and PC/UP, and one was down-accumulated in PI/UP (Table S9). These results showed that compatibility pollination and edible salt treatment might weaken the stigma defense against pathogens, which is similar with the inhibition of SI response. In conclusion, the pollen–stigma response might share some proteins or pathways with plant–pathogen interactions, and edible salt solution treatment destroys the SI of *B. napus* by changing these proteins or pathways.

## 4. Materials and Methods

### 4.1. Plant Materials

One self-compatible line, “Westar”, and two self-incompatible lines, “W-3” and “S-1300”, were used in this study. “W-3” was developed by genetically transforming a pollen self-incompatibility determinant gene *BnSP11-1* into “Westar”, whose self-compatibility came from non-function of a dominant S haplotype *BnSP11-1* by a 3606 bp DNA fragment inserting into the promoter region [71]. The self-incompatibility of “S-1300” was acquired from interspecific hybridization between the *B. napus* line “Huayou8” and the *B. rapa* “Xishuibai” and is determined by the recessive S haplotype S-1300 with high similarity to *BrS-60* [72]. They were grown in the transgenic fields of Huazhong Agricultural University, Wuhan, China from October 2014 to May 2015.

### 4.2. Salt Solution Treatment

Stigmas of fresh flowers of “Westar”, “W-3”, and “S-1300” were treated with edible salt (NaCl ≥ 98.5%) solution. The five concentrations of salt solution were 650 mM, 850 mM, 1050 mM, 1250 mM, and 1450 mM. The stigmas were smeared with salt solution by Chinese writing brushes, and then were artificially pollinated. Pollination with “Westar” pollen grains was taken as one self-compatible control. For “W-3” and “S-1300”, self-incompatible controls were self-pollination and self-pollination after water treatment. Since “Westar” is self-compatible, resulting from the non-functional pollen self-incompatibility determinant gene *BnSP11-1*, its flowers were emasculated one day before anthesis to avoid any contamination by self-pollination. Furthermore, as “Westar” and “W-3” have the same stigma, “Westar” is also incompatible when it pollinates with “W-3” pollen, and so can be taken as self-incompatible control too. Three individual plants each for “Westar”, “W-3”, and “S-1300” were randomly selected as three biological replicates, and all plants were bagged to prevent pollen contamination. For every plant, the major inflorescence was for self-incompatible control, five secondary ramifications were for salt solution treatments, one secondary ramification for self-pollination after water treatment, and one for pollination with “Westar” pollen. Each of the major inflorescences and the secondary ramifications were bagged independently. One week later, the bags were removed to allow seeds to develop naturally. After the seedpods matured, the seeds and flowers produced from each bag were counted, and the self-compatibility index (SCI) as the ratio of the number of seeds to the number of flowers was calculated. At least twenty siliques were sampled for every treatment [73]. Pure NaCl (NaCl ≥ 99.0%, Sinopharm Ltd, Shanghai, China) solution was also tested to eliminate the effect of other compounds of the edible salt solution on propagating SI lines. Statistical analysis was performed by Excel 2013.

### 4.3. Aniline Blue Assay

Aniline blue assay was performed as previously described [71] with minor modifications. Sixteen hours after pollination, *B. napus* pistils were fixed in 3:1 ethanol, glacial acetic acid for 2 h, and softened in 1 N NaOH at 60 °C for 1.5 h. Following softening, the pistils were subjected to washing three times with distilled water, and then stained with basic aniline blue (0.1% aniline blue in 0.1 M K_3_PO_4_) for 2.5 h. The stained pistils were gently put onto a microscopic slide glass and gently squashed by the cover glass. Samples were observed under a fluorescence microscope (Ax 10, Zeiss, Jena, Germany). At least six pistils were observed for every pollination treatment.

### 4.4. Stigma Collection and Protein Sample Preparation

“Westar” flowers were emasculated one day before anthesis. Stigmas were unpollinated (UP), pollinated with “Westar” pollen (PC), pollinated with “W-3” pollen (PI), and pollinated with “W-3” pollen after edible salt solution (1050 mM) treatment (NA). Thirty minutes after pollination, stigmas of each sample were harvested by cutting the pistil just below the base of the stigma, immediately frozen in liquid nitrogen, and stored at −80 °C. Each sample had three biological replicates, and the stigmas of every biological replicate came from at least three individual plants.

Proteomics analysis was carried out at BGI (Shenzhen, China). Two independent biological replicates were performed. For each biological replicate, approximately 1500 stigmas were ground into powder in liquid nitrogen, extracted with lysis buffer (7 M urea, 2 M thiourea, 4% CHAPS, 40 mM Tris-HCl, pH 8.5) containing 1 mM PMSF and 2 mM EDTA (final concentration). After 5 min, 10 mM DTT (final concentration) was added to the samples. The suspension was sonicated at 200 W for 15 min, and then centrifuged at 4 °C, 30,000*g* for 15 min. The supernatant was mixed well with a 5× volume of chilled acetone containing 10% (*v*/*v*) TCA, and incubated at −20 °C overnight. After centrifugation at 4 °C, 30,000*g*, the supernatant was discarded. The precipitate was washed three times with chilled acetone. The pellet was air-dried and dissolved in lysis buffer (7 M urea, 2 M thiourea, 4% NP40, 20 mM Tris-HCl, pH 8.0–8.5). The suspension was sonicated at 200 W for 15 min and centrifuged at 4 °C, 30,000*g* for 15 min. The supernatant was transferred to a new centrifuge tube, and 10 mM DTT was added (final concentration), followed by incubation at 56 °C for 1 h to reduce disulfide bonds of protein species. After that, 55 mM IAM (SIGMA-ALDRICH, Burlington, MA, USA) was added (final concentration) to inhibit cysteines, and incubated in a darkroom for 1 h. To precipitate protein species, a 5× volume of chilled acetone was mixed well with the supernatant, and incubated for 2 h at −20 °C. The mixture was centrifuged at 4 °C for 3000× *g*, the supernatant was discarded, and the pellet was dried in air for 5 min. The pellet was dissolved in 500 µL, 0.5 M TEAB (Applied Biosystems, Milan, Italy) and sonicated for 15 min at 200 W. Finally, centrifuged the mixture at 4 °C, 3000× *g* for 15 min. The supernatant was transferred to a new tube, and the protein concentration was quantified with a Bio-Rad protein assay kit (Bio-rad, Hercules, CA, USA) based on the Bradford method using BSA as a standard. The proteins in the supernatant were kept at −80 °C until further analysis.

### 4.5. iTRAQ Labeling and SCX Fractionation

Total protein (100 µg) was collected from each sample solution, and the protein was then digested with Trypsin Gold (Promega, Madison, WI, USA) at a 30:1 ratio of protein/trypsin at 37 °C for 16 h. After trypsin digestion, peptides were dried by vacuum centrifugation. The peptides were reconstituted in 0.5 M TEAB, and processed according to the manufacturer’s protocol for the 8-plex iTRAQ reagent (Applied Biosystems). Briefly, one unit of iTRAQ reagent was thawed and reconstituted in 24 µL isopropanol. Samples were labeled with the iTRAQ tags, and the tag for each sample is shown in Figure S3. The peptides were labeled with the isobaric tags and incubated at room temperature for 2 h. Peptides were labeled with iTRAQ reagents 113 and 115 for UP, 114 and 116 for PC, 117 and 119 for PI, and 118 and 121 for NA. By vacuum centrifugation, the mixtures of labeled peptide were pooled and dried. An LC-20AB HPLC Pump system (Shimadzu, Kyoto, Japan) was used to perform SCX chromatography. To reconstitute the iTRAQ labeled peptide mixtures, 4 mL buffer A (25 mM NaH_2_PO_4_ in 25% ACN, pH 2.7) was added, and loaded on a 4.6 × 250 mm Ultremex SCX column containing 5 µm particles (Phenomenex, Torrance, CA, USA). The peptide mixtures were eluted in a gradient of buffer A for 10 min, 5–60% buffer B (25 mM NaH_2_PO_4_, 1 M KCl in 25% ACN, pH 2.7) for 27 min, 60–100% buffer B for 1 min, at a flow rate of 1 mL/min. Before equilibrating with buffer A for 10 min prior to the next injection, the system was maintained at 100% buffer B for 1 min. By measuring the absorbance at 214 nm to monitor the elution results, and fractions were collected every 1 min. Finally, the eluted peptides pooled into 20 fractions, then the fractions were desalted with a Strata X C18 column (Phenomenex, Torrance, CA, USA) and vacuum-dried.

### 4.6. LC-MS/MS Measurement and Data Analysis

Each fraction was resuspended in buffer A (5% ACN, 0.1% FA) and centrifuged for 10 min at 20,000*g*; the final average concentration of peptide was approximately 0.5 µg/µL. Supernatant (10 µL) was loaded on an LC-20AD nano HPLC (Shimazu, Kyoto, Japan) by autosampler onto a 2 cm C18 trap column. The fractions were eluted on a 10 cm analytical C18 column (inner diameter 75 µm) that was packed in-house. The peptides were loaded at 8µL/min for 4 min, the 35 min gradient was run at 300 nL/min from 2% to 35% B (95% ACN, 0.1% FA), then a 5 min linear gradient to 60%, a 2 min linear gradient to 80%, with maintenance at 80% B for 4 min, and finally, a return to 5% in 1 min. Data were acquired using a Triple TOF 5600 System fitted with a Nanospray III source (AB SCIEX, Concord, ON, USA) and a pulled quartz tip as the emitter (New Objectives, Woburn, MA, USA). Data acquisition was operated with an ion spray voltage of 2.5 kV, curtain gas of 30 PSI, nebulizer gas of 15 PSI, and an interface heater temperature of 150 °C. The MS was performed with an resolving power (RP) greater than or equal to 30,000 FWHM for TOF MS scans. For information dependent acquisition (IDA), survey scan acquisitions were operated for 250 ms with a 2+ to 5+ charge-state, and if exceeding a threshold of 120 counts per second (counts/s), as many as 30 product ion scans were collected. The total cycle time was set to 3.3 s. The Q2 transmission window was 100 Da for 100%. Each scan summed 4 time bins at a pulser frequency value of 11 kHz by monitoring of the 40 GHz multichannel TDC detector with a four-anode channel detection ion. All precursor ions for collision-induced dissociation applied a sweeping collision energy setting of 35 ± 5 eV coupled with iTRAQ adjust rolling collision energy. Dynamic exclusion was set as 1/2 of peak width (15 s), and then the precursor refreshed from the exclusion list. Raw data files were acquired from Orbitrap, which were converted into MGF files with Proteome Discoverer 1.2 (PD 1.2, Thermo), (5600 msconverter, Waltham, MA, USA) and the files were searched. Protein identification was operated using a Mascot search engine (version 2.3.02, Matrix Science, London, UK) against a database containing *Brassica napus* sequences (http://www.genoscope.cns.fr/brassicanapus/). For protein identification, a mass tolerance of 0.05 Da was the allowance for intact peptide masses, and ±0.1 Da for fragmented ions, permitting only one missed cleavage in the trypsin digests. Set Gln->pyro-Glu (N-term Q), Oxidation (M), and Deamidated (NQ) were set as the potential variable modifications, and Carbamidomethyl (C), iTRAQ 8plex (N-term), and iTRAQ 8plex (K) were set as fixed modifications. The charge states of peptides were set to +2 and +3. In particularly, an automatic decoy database search was performed in Mascot, where the decoy checkbox was chosen with a random sequence of database generated and tested for raw spectra, as well as the real database. To reduce the probability of false peptide identification, only these peptides were counted as identified, which were greater than “identity” with the 95% confidence interval by a Mascot probability analysis. Each confident protein identification used at least one unique peptide. A protein containing at least two unique spectra was required for protein quantitation. Then, protein ratios were weighted and normalized by the median ratio in Mascot. Protein ratios with *p*-values < 0.05, and fold changes of >1.3 or <0.77 were considered significant. Functional classifications of the protein species were conducted with the Blast2GO program against the non-redundant protein database (NR; NCBI). All identified protein species were classified and grouped by using KEGG database (http://www.genome.jp/kegg/) and COG database (http://www.ncbi.nlm.nih.gov/COG/).

### 4.7. Real-Time Quantitative Reverse Transcription PCR (qPCR)

Total RNA from three biological replicates was isolated using a plant mini RNeasy kit (Qiagen, Hilden, Germany). After RNA quality and quantity measurement, five micrograms of RNA was DNase-treated using a DNA-free kit (Ambion, http://www.ambion.com), and first-strand cDNA synthesis and qPCR were carried out as previously described [74]. Gene-specific primers used for qPCR were designed with Primer Premier 5 software (Palo Alto, CA, USA) according to *B. napus* cDNA sequences (Table S10). Actin (GenBank accession no.: AF111812) was used as an internal control to normalize transcript levels for all the expression analyses.

## 5. Conclusions

Our study showed that iTRAQ was a powerful tool for quantitative proteomics analysis of salt-induced self-compatibility in *B. napus*. A large number of differentially accumulated proteins were identified, including 307 proteins in UP/NA. Further bioinformatics analysis indicated that the DAPs were involved in ribosome, endocytosis, proteasome, plant–pathogen interaction, phagosome, and ubiquitin-mediated proteolysis pathway. Upregulated expression of SC-related proteins may be required for salt-solution destruction of SI, and may help plants facilitate SC responses. Knockout or overexpression of the DAPs in NA/UP would identify genes responsible for salt-induced self-compatibility. In summary, our results showed comprehensive extractome coverage of salt-induced self-compatibility, and the potential candidate proteins may provide a starting point in the elucidation of the molecular mechanisms and provide potential genetic resources for the breeding of self-incompatibility lines.

## Figures and Tables

**Table 1:** KEGG pathway enrichment analysis of differentially accumulated proteins based on the Venn diagram.

Categories of Proteins	Number of Proteins	Pathway	Pathway ID	*p*-Value
PC/UP unique DAPs	194	Photosynthesis	ko00195	0.0023
		Amino sugar and nucleotide sugar metabolism	ko00520	0.0028
		Proteasome	ko03050	0.0036
		Metabolic pathways	ko01100	0.0063
		Cyanoamino acid metabolism	ko00460	0.0066
		Oxidative phosphorylation	ko00190	0.019
		Carbon fixation in photosynthetic organisms	ko00710	0.0224
PI/UP unique DAPs	71	Inositol phosphate metabolism	ko00562	0.042
		Biosynthesis of secondary metabolites	ko01110	0.0457
NA/UP unique DAPs	134	Ascorbate and aldarate metabolism	ko00053	0.0094
		Ribosome	ko03010	0.0099
		Plant–pathogen interaction	ko04626	0.0213
		Phagosome	ko04145	0.0259
PC/UP_NA/UP intersection DAPs	94	Ribosome	ko03010	5.46 × 10^−13^
		Phagosome	ko04145	0.0202
PC/UP_PI/UP intersection DAPs	39	Glyoxylate and dicarboxylate metabolism	ko00630	0.0133
		Alanine, aspartate, and glutamate metabolism	ko00250	0.0434
PI/UP_NA/UP intersection DAPs	17	Glycine, serine, and threonine metabolism	ko00260	0.0177
		Biosynthesis of amino acids	ko01230	0.0273
		Biotin metabolism	ko00780	0.0417
		Metabolic pathways	ko01100	0.0478
PC/UP_PI/UP_NA/UP intersection DAPs	62	Oxidative phosphorylation	ko00190	0.0129
		Photosynthesis	ko00195	0.0138
		Valine, leucine, and isoleucine degradation	ko00280	0.0138
		Plant–pathogen interaction	ko04626	0.0223

## Supplementary Materials

Supplementary materials can be found at http://www.mdpi.com/1422-0067/19/6/1652/s1.
