# Supplementary material for: Mechanism of Salt-Induced Self-Compatibility Dissected by Comparative Proteomic Analysis in Brassica napus L
Source: Int J Mol Sci. 2018 Jun 3;19(6):1652. doi: 10.3390/ijms19061652 (PMC6032146; doi:10.3390/ijms19061652)
Supplement: Supplementary file 1 [file ijms-19-01652-s001.zip › ijms-30527suppl/Supplementary Figures.docx]

**Mechanism of salt-induced self-compatibility dissected by comparative proteomic analysis in** ***Brassica napus L.***

Yong Yang^1^, Zhiquan Liu^1^, Tong Zhang^1^, Guilong Zhou^1^, Zhiqiang Duan^1^, Bing Li^1^, Shengwei Dou^1^, Xiaomei Liang^1^, Jinxing Tu^1^, Jinxiong Shen^1^, Bin Yi^1^, Tingdong Fu^1^, Cheng Dai^1*^ and Chaozhi Ma^1*^

^1^National Key Laboratory of Crop Genetic Improvement, National Center of Rapeseed Improvement in Wuhan, Huazhong Agricultural University, Wuhan 430070, P.R. China

**Table S1** All identified proteins in this study.

**Table S2** Significantly differentially accumulated proteins (DAPs) in UP-VS-PC, UP-VS-PI and UP-VS-NA.

**Table S3** The classification of differentially accumulated proteins (DAPs) based on venn diagram.

**Table S4** GO enrichment analysis differentially accumulated proteins (DAPs) of UP-VS-PC, UP-VS-PI and UP-VS-NA in biological process.

**Table S5** GO enrichment analysis of the commonly changed proteins between UP-VS-PC and UP-VS-NA in biological process.

**Table S6** Differentially accumulated proteins (DAPs) enriched in response to salt stress between UP-VS-PC and UP-VS-NA.

**Table S7** KEGG analysis of differentially accumulated proteins (DAPs) in UP-VS-PC, UP-VS-PI and UP-VS-NA.

**Table S8** The differentially accumulated proteins (DAPs) involved in candidate pathway in UP-VS-PC, UP-VS-PI and UP-VS-NA.

**Table S9** KEGG enrichment analysis of the classification differentially accumulated proteins (DAPs) based on venn diagram.

**Table S10** Specific primer pairs used for qRT-PCR expression analysis.


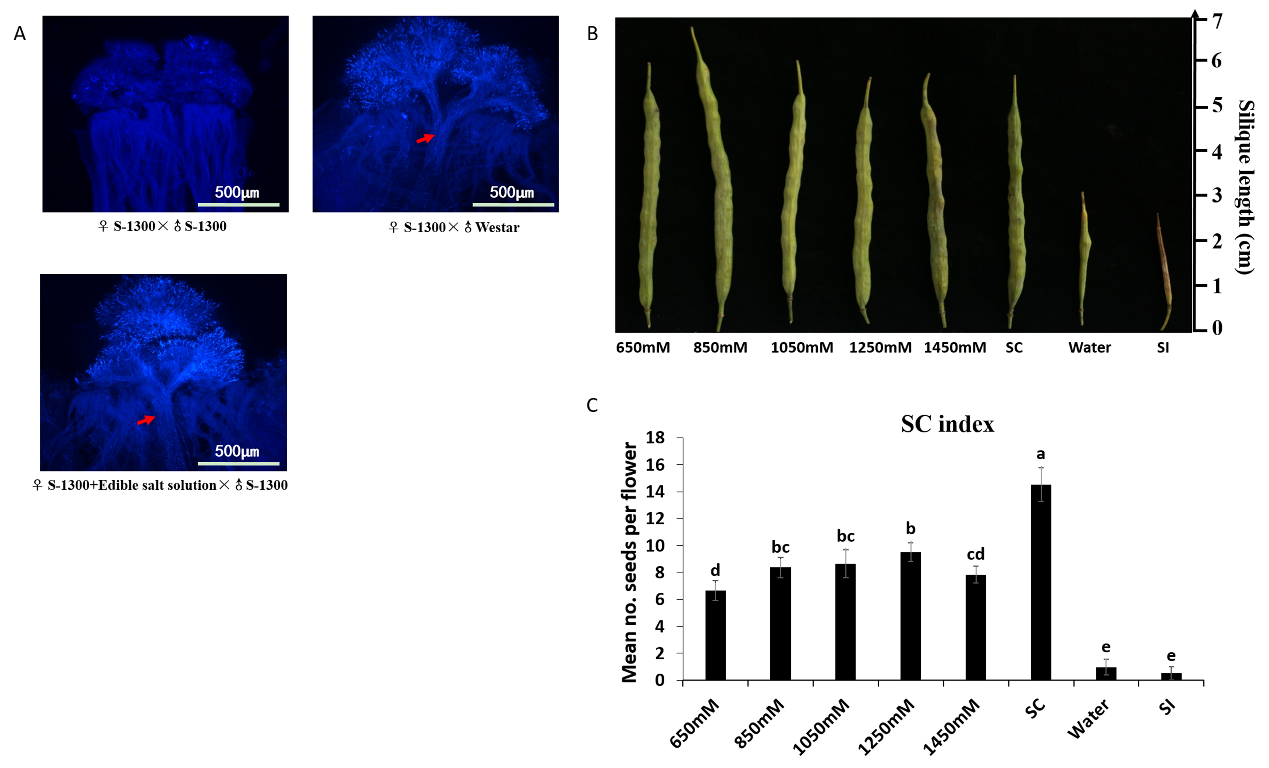


**Figure S1.** Breakdown of self-incompatibility by edible salt solution in S-1300. Pollination with ‘Westar’ pollen grains was taken as self-compatibility control (SC); self-incompatibility control were pollination with self-pollen grains (SI) and pollination with self-pollen grains after water treatment (Water); Concentrations of edible salt solution were 650mM, 850mM, 1050mM, 1250mM and 1450mM. **(A)** Aniline blue assays performed 16 h after pollination with self-pollen grains, compatible ‘Westar’ pollen grains and self-pollen grains after 1050mM edible salt solution treatment on the stigmas of S-1300. Every treatment included at least six individual pistils. Red arrows indicated abundant pollen tubes. **(B)** Mature pods developed from S-1300 pistils that pollinated with self-pollen grains after treated by edible salt solutions. **(C)** Mean self-compatibility indexes of S-1300 after edible salt solution treatment, and their statistic test by one-way ANOVA with Scheffe post hoc tests. The different letters represent means that are significantly different at P<0.05; Error bars mean standard deviation.


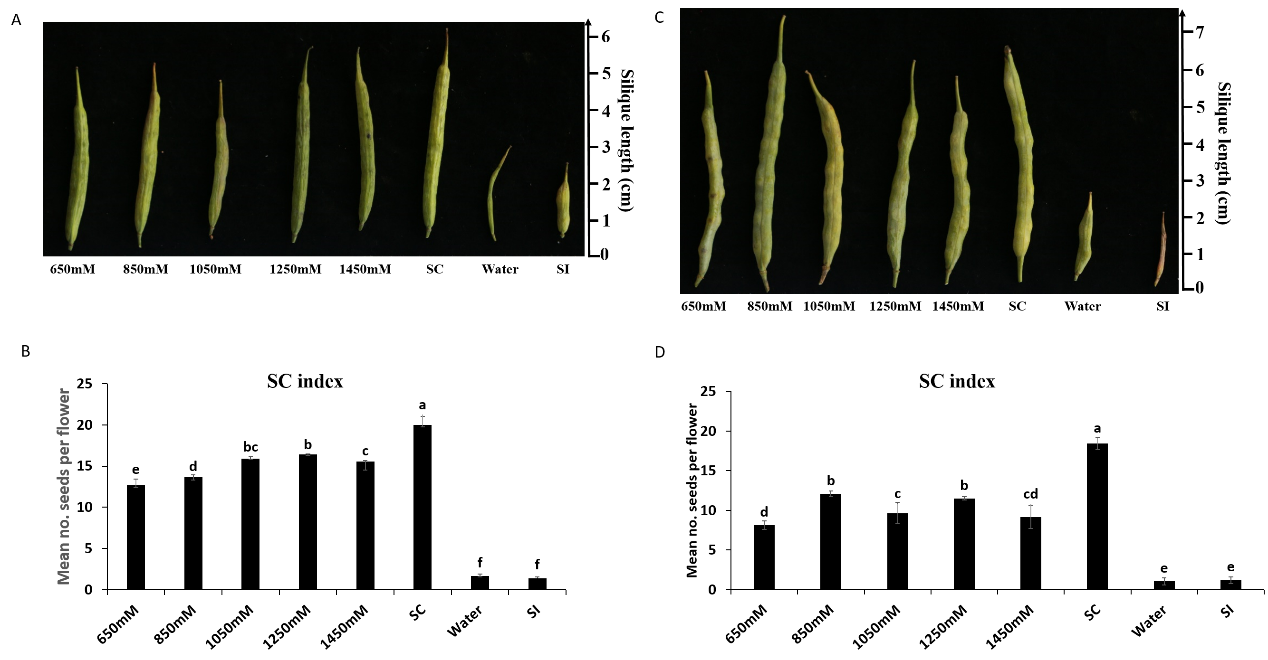


**Figure S2.** Breakdown of self-incompatibility by NaCl solution in ‘W-3’ and S-1300. Pollination with ‘Westar’ pollen grains was taken as self-compatibility control (SC); self-incompatibility control were pollination with self-pollen grains (SI) and pollination with self-pollen grains after water treatment (Water); Concentrations of NaCl solution were 650mM, 850mM, 1050mM, 1250mM and 1450mM. **(A, C)** Mature pods developed from ‘W-3’ and S-1300 pistils that pollinated with self-pollen grains after treated by NaCl solutions. **(C)** Mean self-compatibility indexes of ‘W-3’ and S-1300 after NaCl solution treatment, and their statistic test by one-way ANOVA with Scheffe post hoc tests. The different letters represent means that are significantly different at P<0.05; Error bars mean standard deviation.


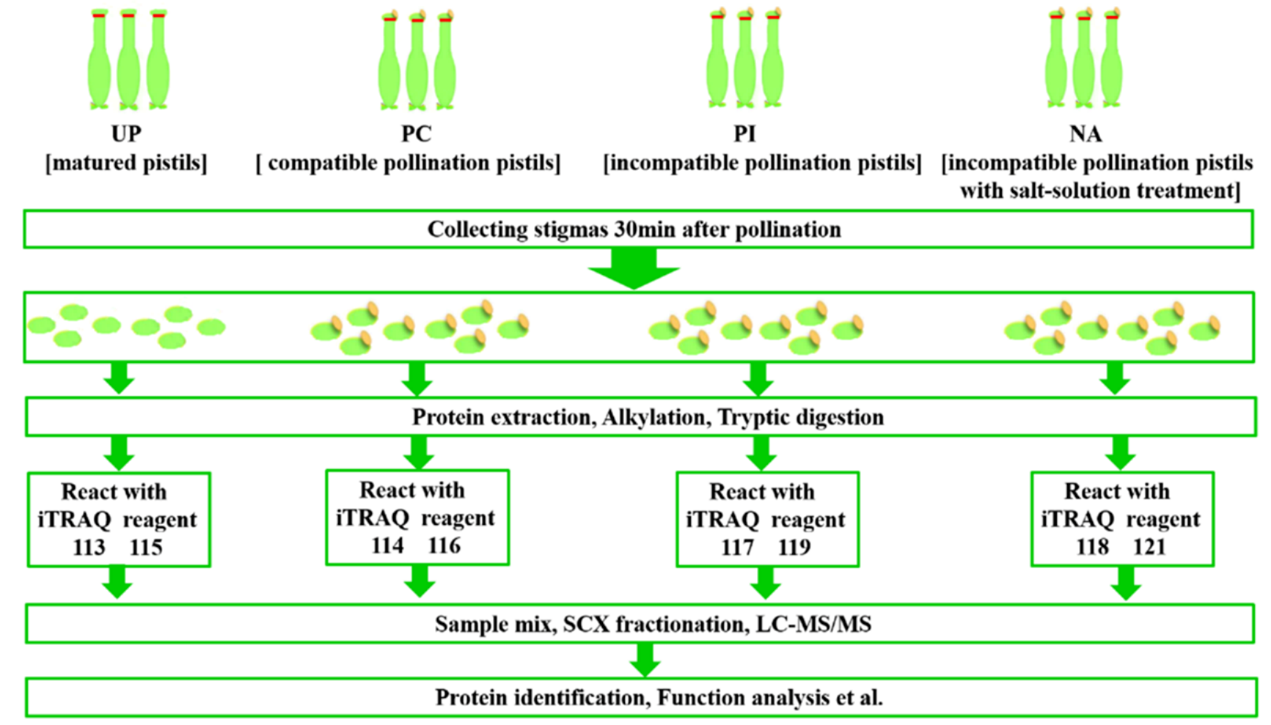


**Figure S3.** Workflow for characterization of pollen-stigma interaction mechanisms of different pollination treatments using iTRAQ. ‘Westar’ flowers, which were emasculated one day before flowers opening, were pollinated with no pollen grains (UP), ‘Westar’ pollen grains (PC), transgenic ‘W-3’ pollen grains (PI) and transgenic ‘W-3llen grains after edible salt solution treatment (NA). Stigmas were collected by cutting the pistil just below the base of the stigma (red line indicated) at 30min after pollination and immediately frozen in liquid nitrogen and stored at -80 ℃ until used. Extracted proteins were purified, reductive alkylation, tryptic digestion and labeled with iTRAQ. Labeled peptides were separated by SCX chromatography and fractions were analyzed by reversed phase LC-MS/MS. Finally, bioinformatics analysis was performed.


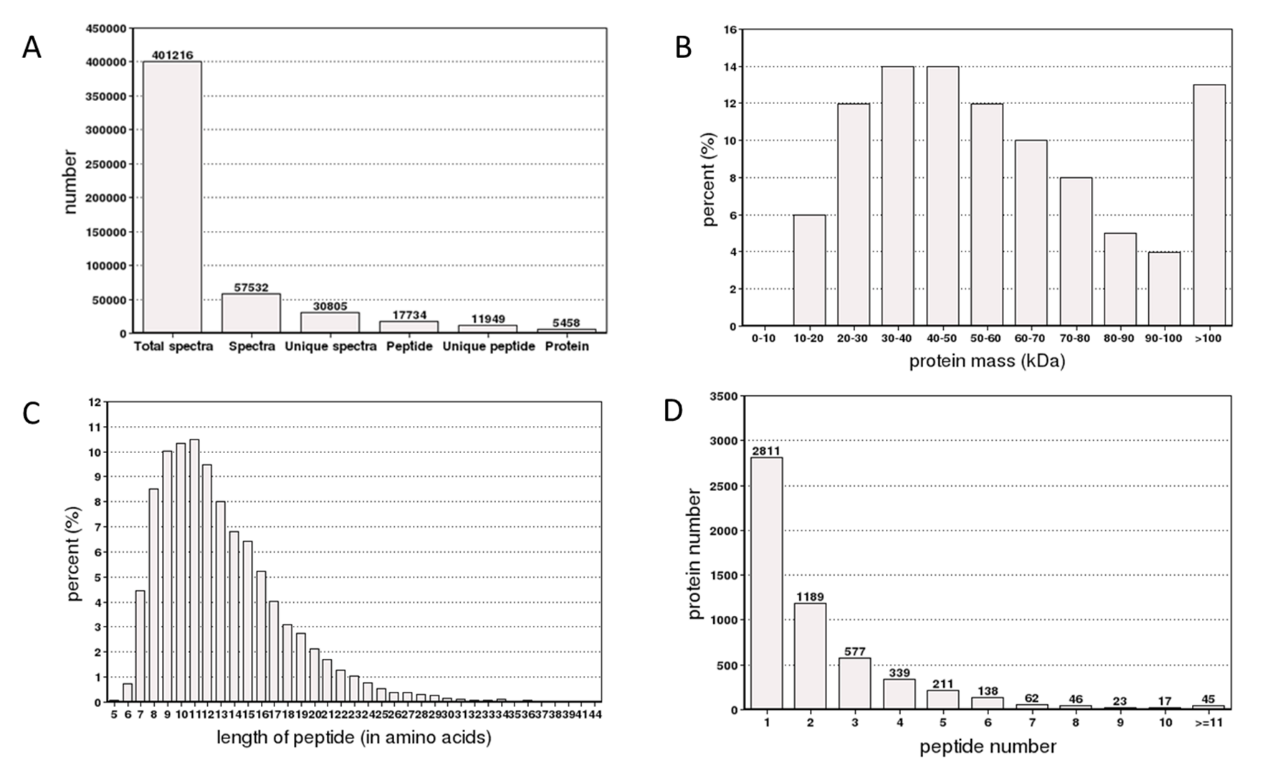


**Figure S4.** Basic data generated by iTRAQ. **(A)** Basic information statistics. “Total Spectra” means the number of all the spectra spectrograms identified by iTRAQ. “Spectra” means the number of identified spectra spectrograms. “Unique Spectra” means the number of spectra spectrograms matching the unique peptide sequences. “Peptide” means the number of detected peptide sequences. “Unique Peptide” means the number of unique peptide sequences in the identified proteins. “Protein” means the number of identified proteins. **(B)** Protein mass distribution. The numbers of proteins distributed in different rang of molecular mass were shown. **(C)** Peptide length distribution. The numbers of proteins distributed in different rang of peptide sequence coverage were shown. **(D)** Peptide number distribution. Based on the number of matched peptides, the identified proteins were grouped. The numbers of proteins in each group were showed.


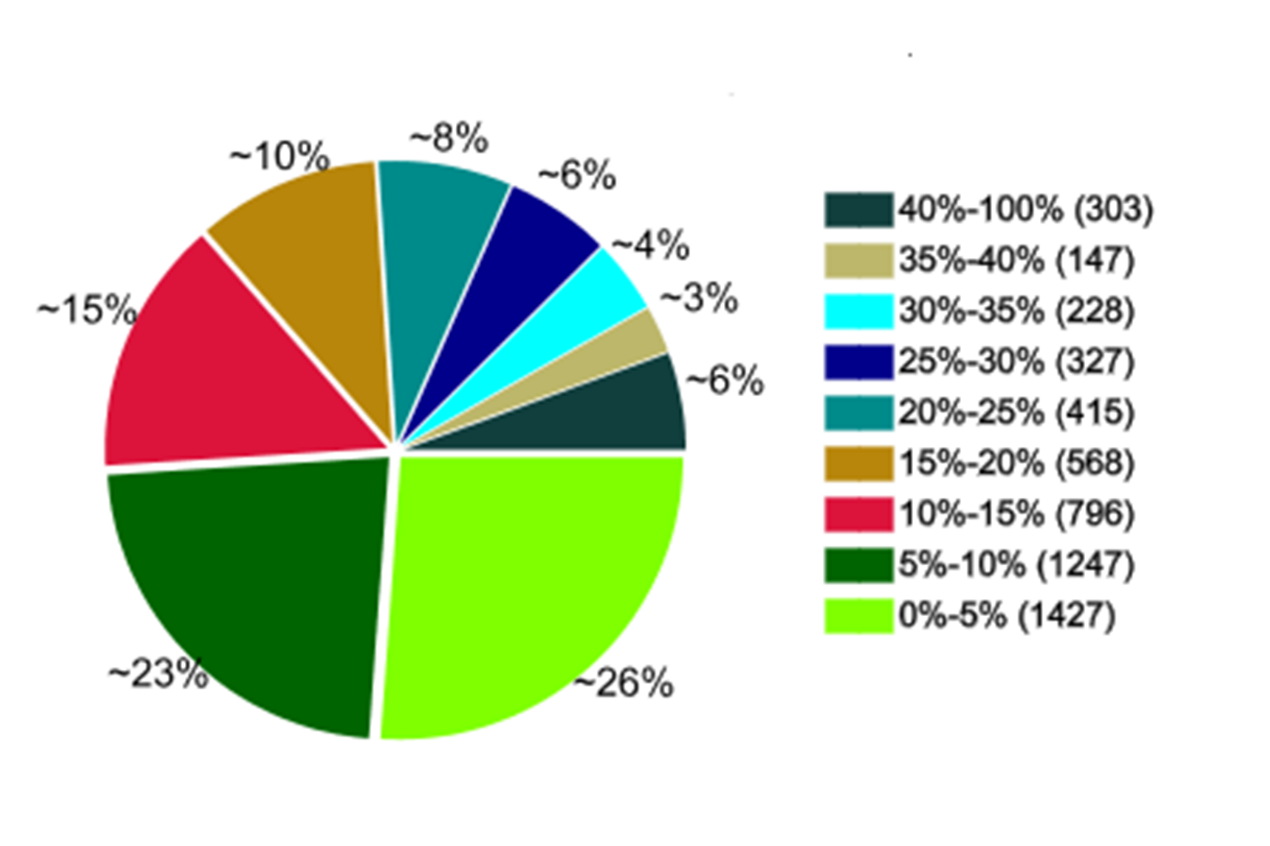


**Figure S5.** The distribution of protein sequence coverage.

**
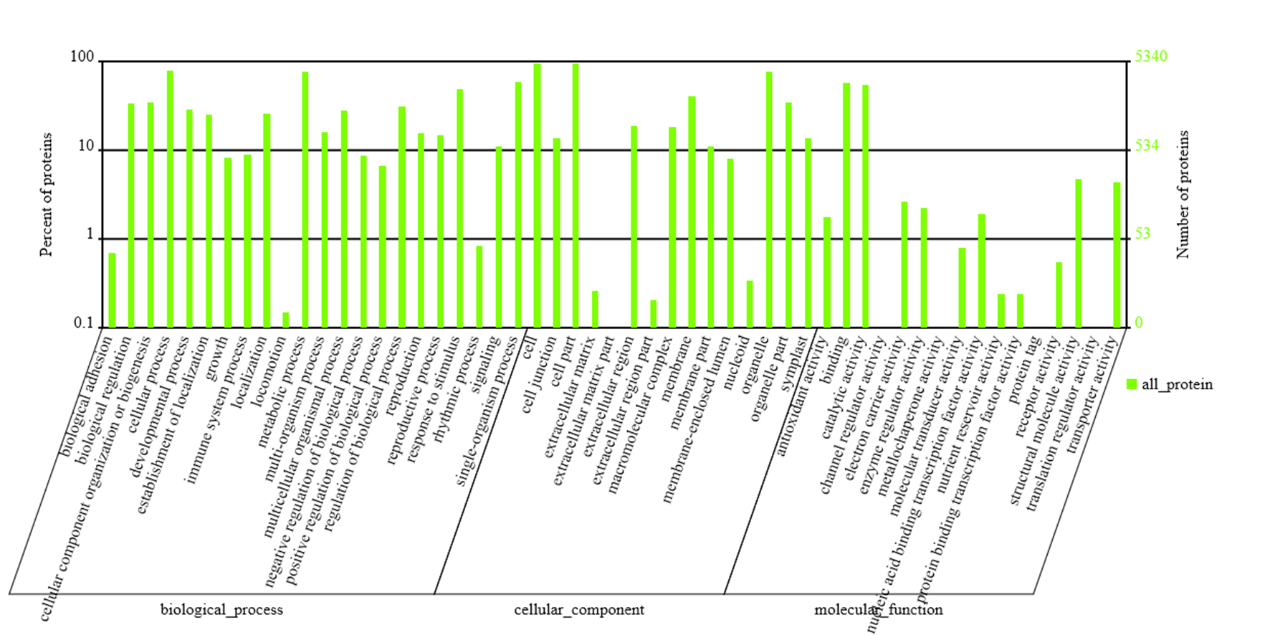
**

**Figure S6.** GO annotation of all identified proteins.

**
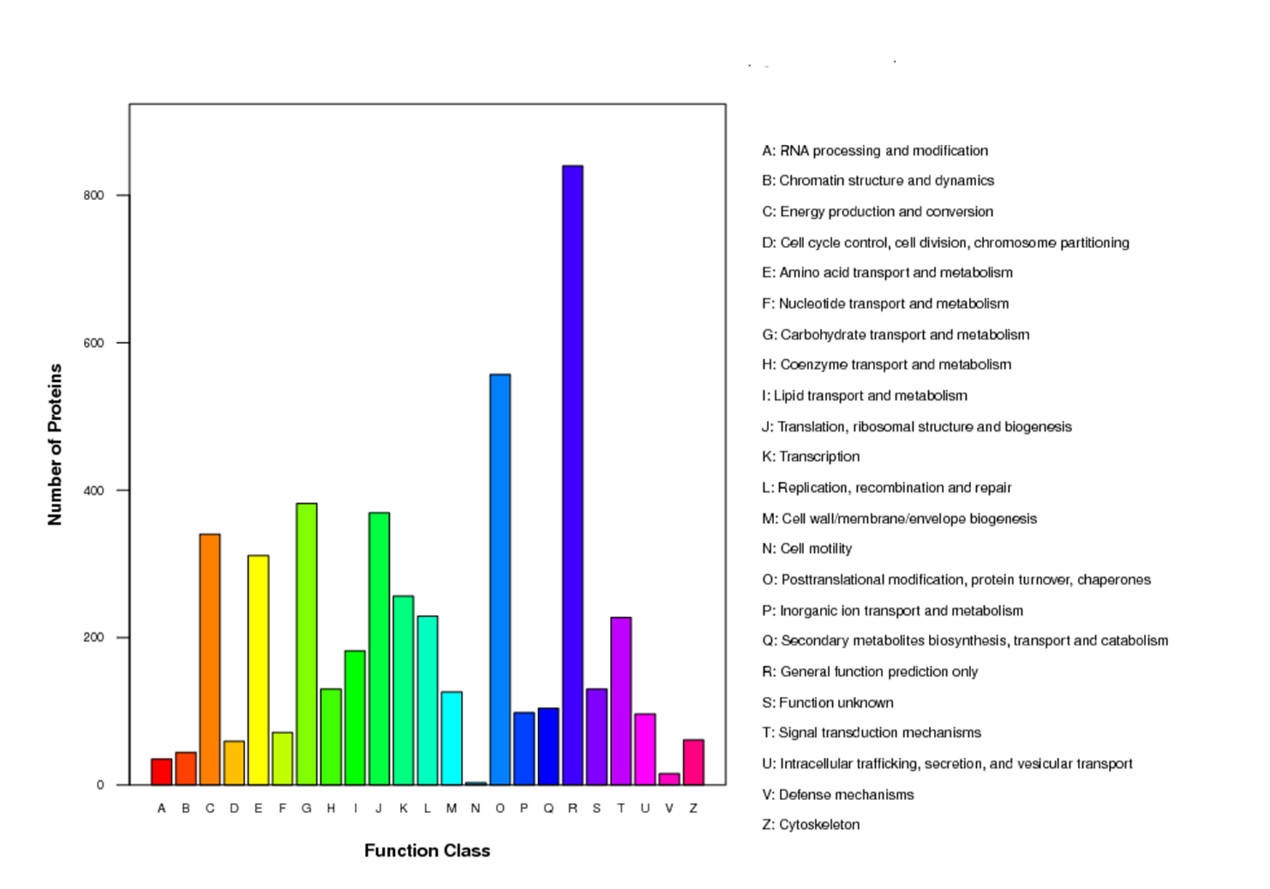
**

**Figure S7.** COG function analysis of all identified proteins.


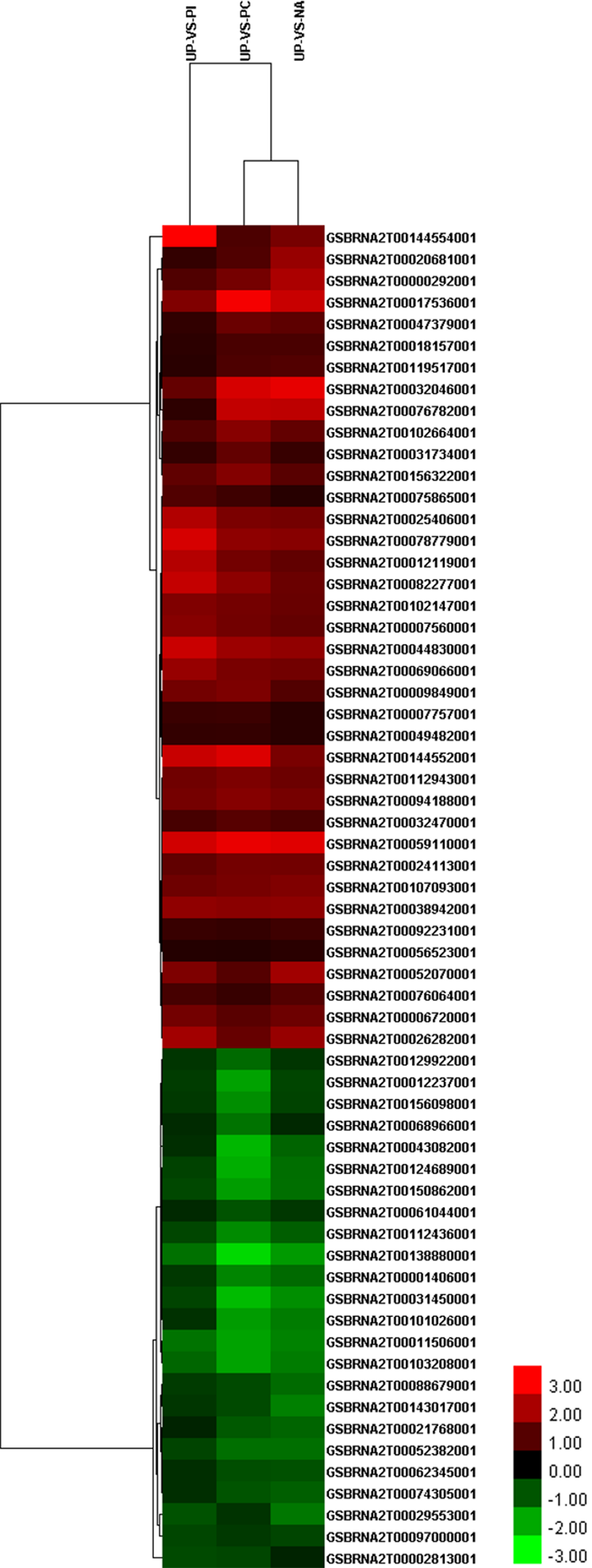


**Figure S8.** Cluster analysis of 62 commonly differentially accumulated proteins among UP-VS-PC, UP-VS-PI and UP-VS-NA.


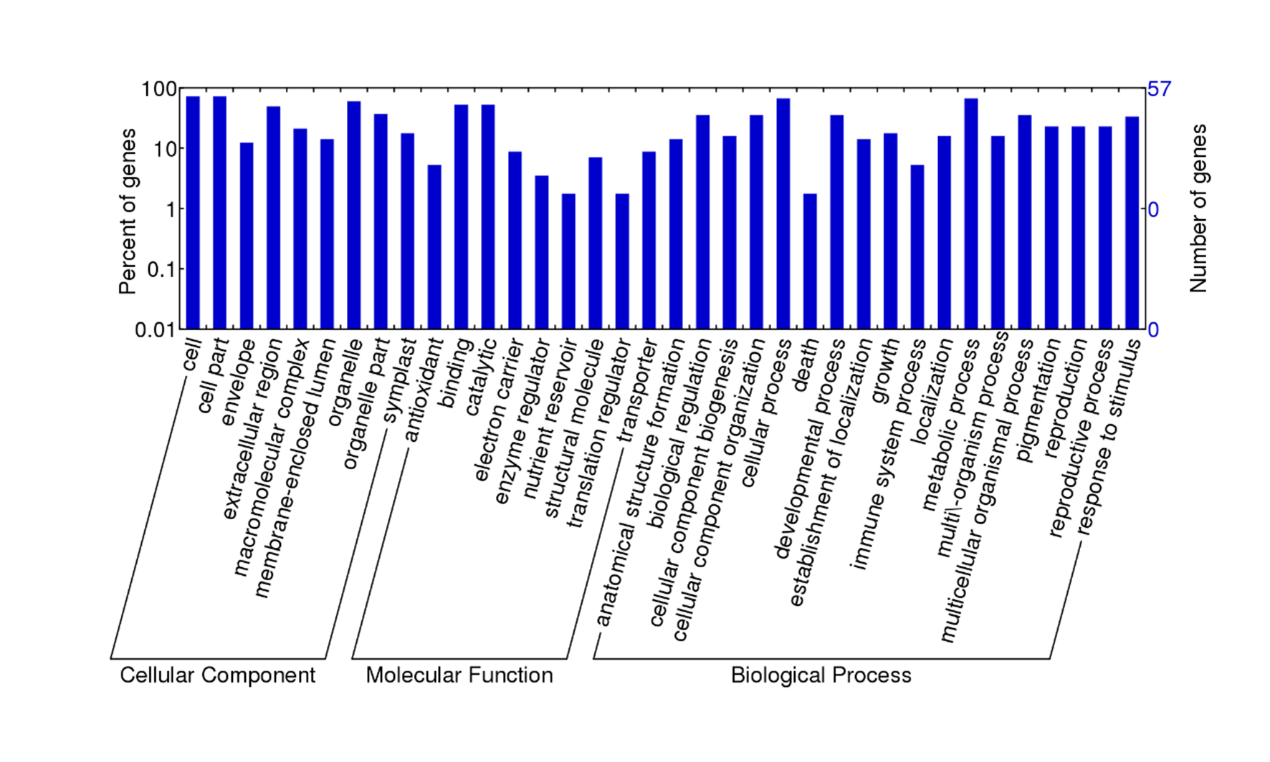


**Figure S9.** GO function analysis of the 94 commonly changed proteins between UP-VS-PC and UP-VS-NA.


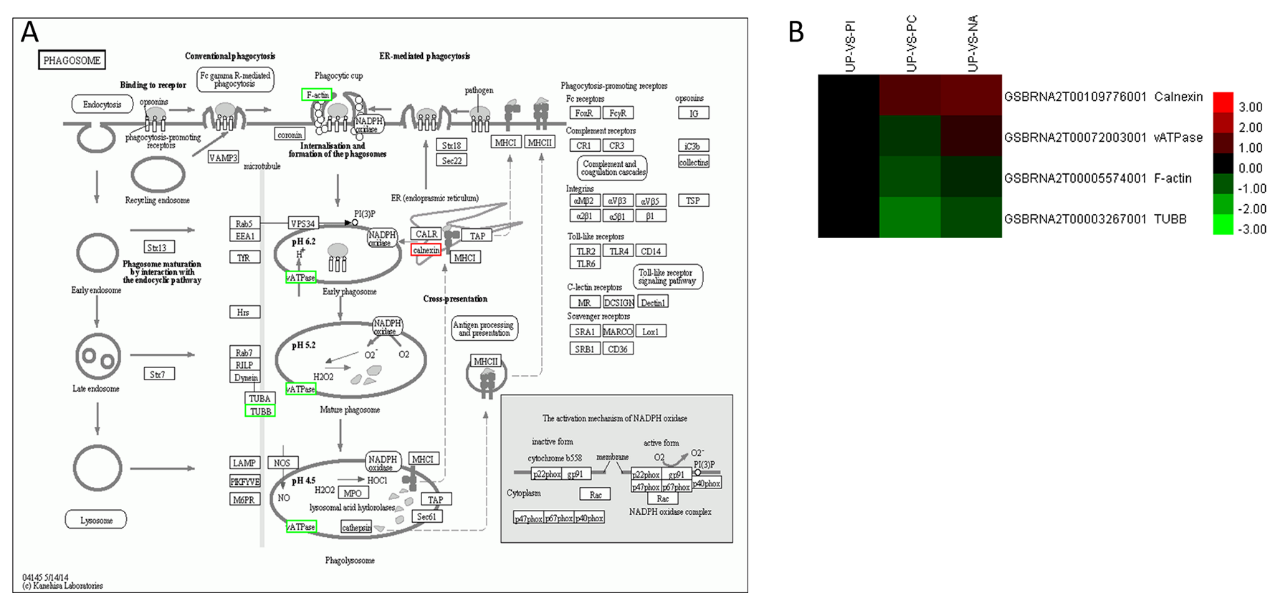


**Figure S10.** The overlapping differentially accumulated proteins between UP-VS-PC and UP-VS-NA enriched in the phagosome. **(A)** Four protein species mapped to phagosome. **(B)** Heat map of the four differentially accumulated proteins, and red indicates up-accumulated and green indicates down-accumulated.
